# Supplementary figures and images for: DTX3L ubiquitin ligase ubiquitinates single-stranded nucleic acids
Source: eLife. 2024 Oct 8;13:RP98070. doi: 10.7554/eLife.98070 (PMC11460948; doi:10.7554/eLife.98070)

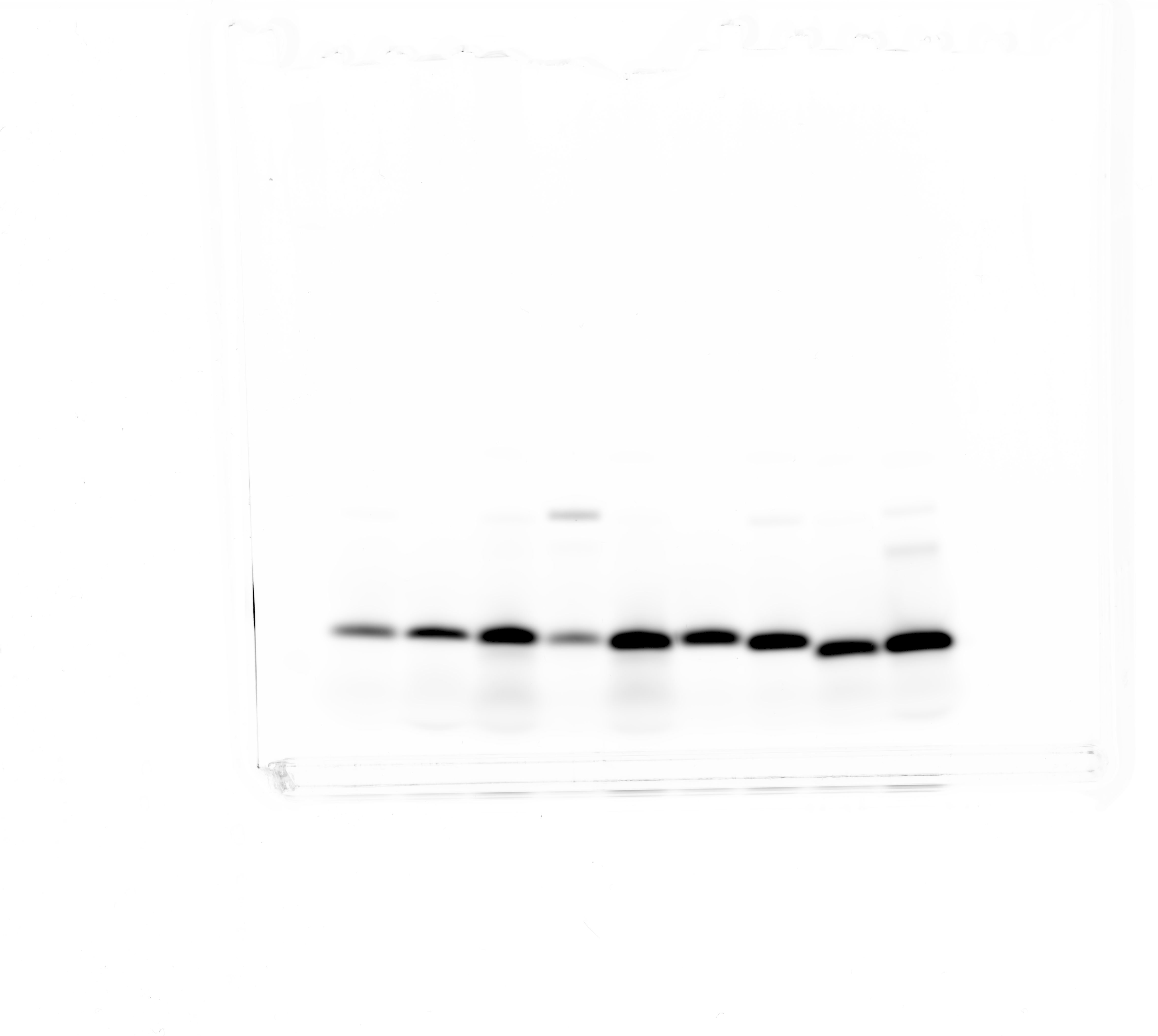

Supplement: Figure 1—source data 3. [file elife-98070-fig1-data3.zip › Figure 1_source data 3/1F.tif]

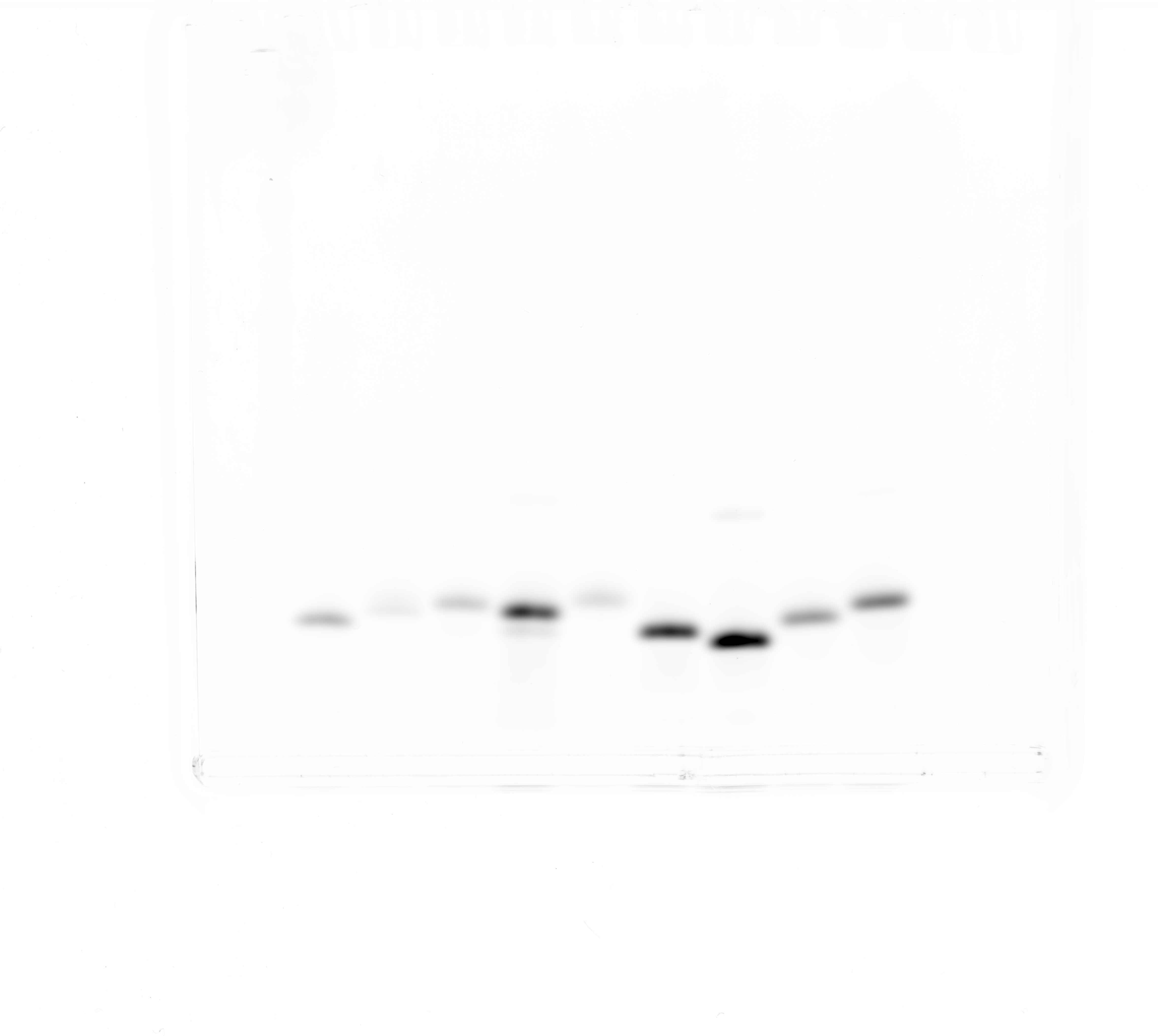

Supplement: Figure 1—source data 3. [file elife-98070-fig1-data3.zip › Figure 1_source data 3/1G.tif]

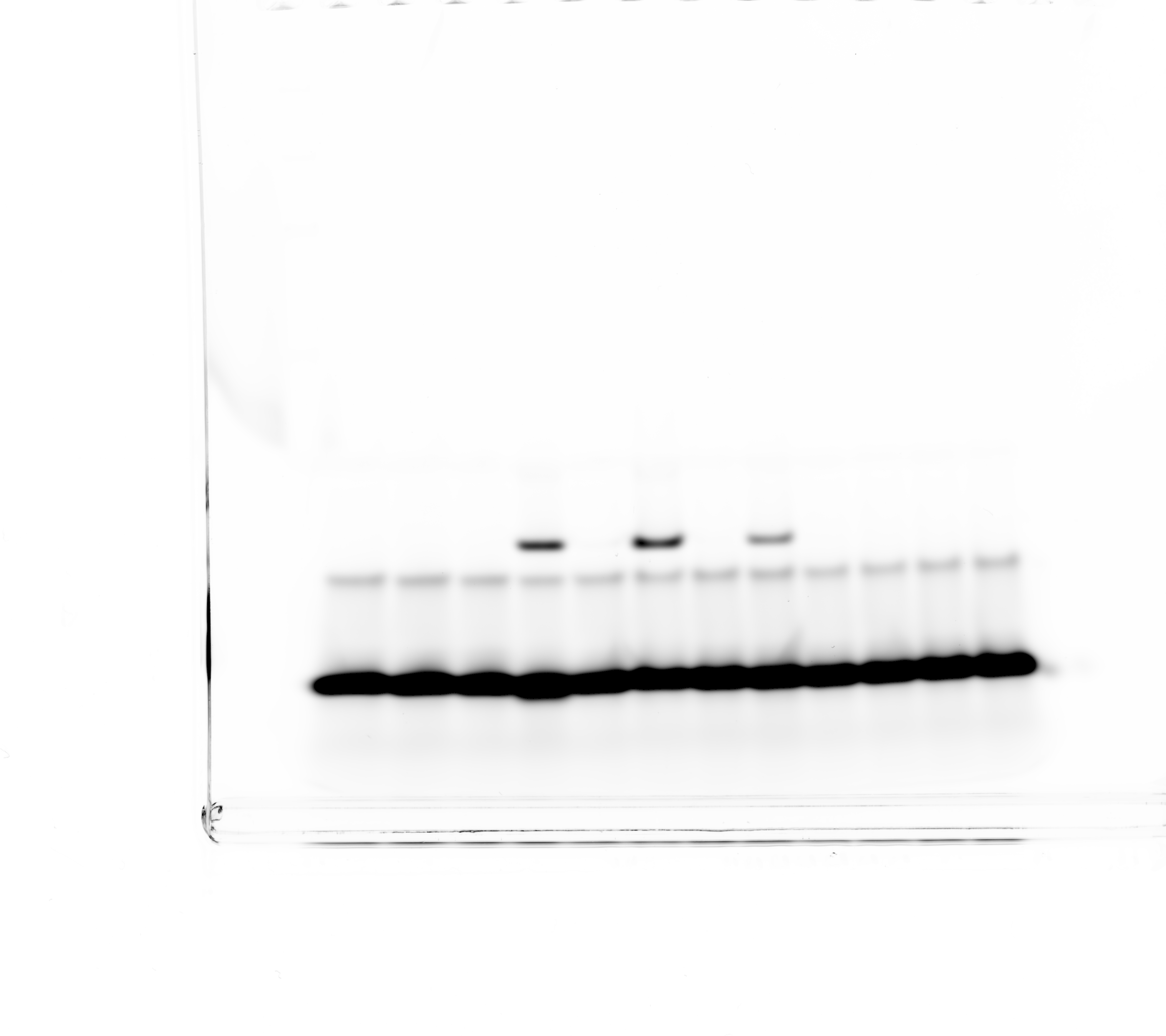

Supplement: Figure 1—source data 3. [file elife-98070-fig1-data3.zip › Figure 1_source data 3/1H.tif]

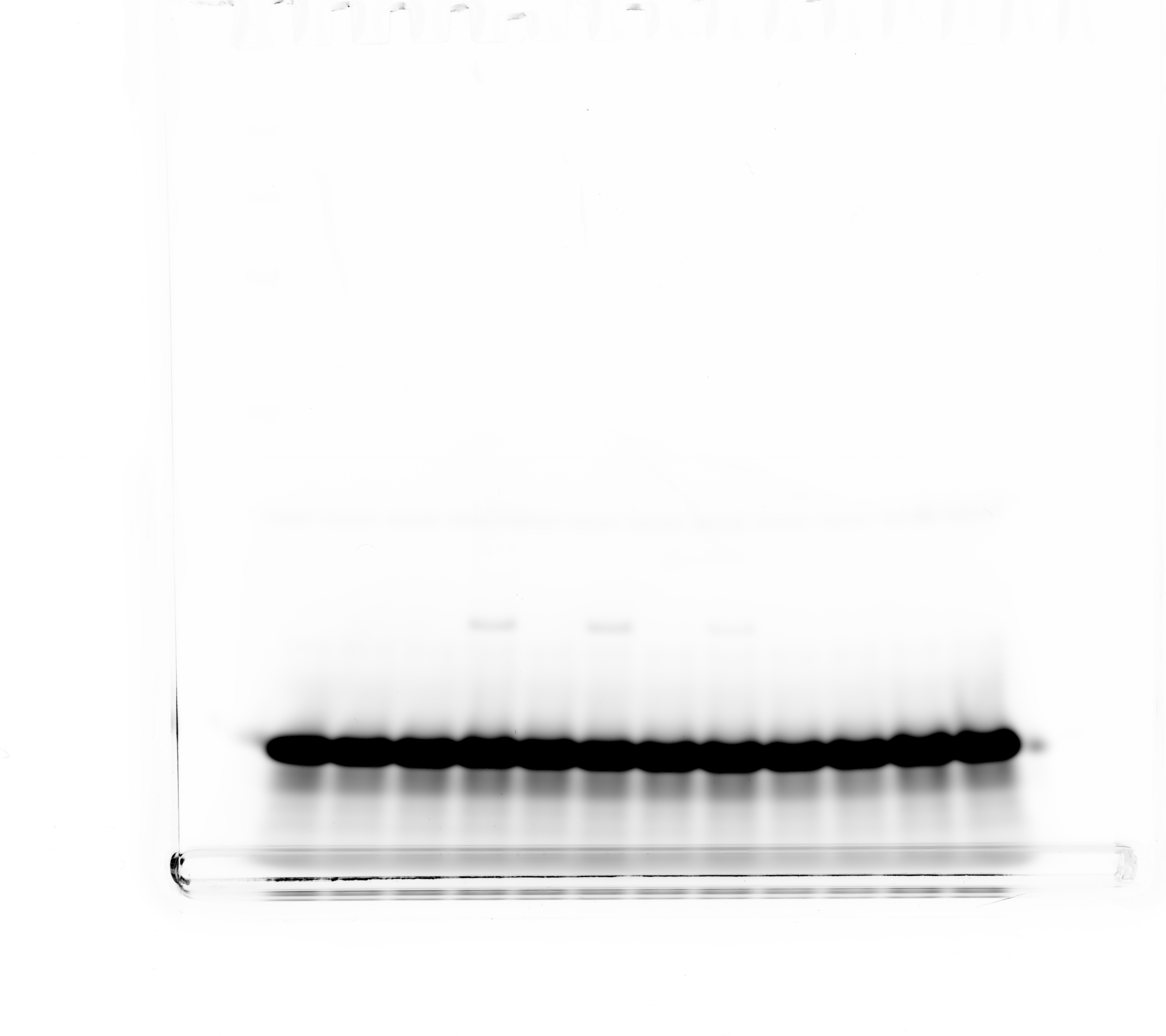

Supplement: Figure 1—source data 3. [file elife-98070-fig1-data3.zip › Figure 1_source data 3/1I.tif]

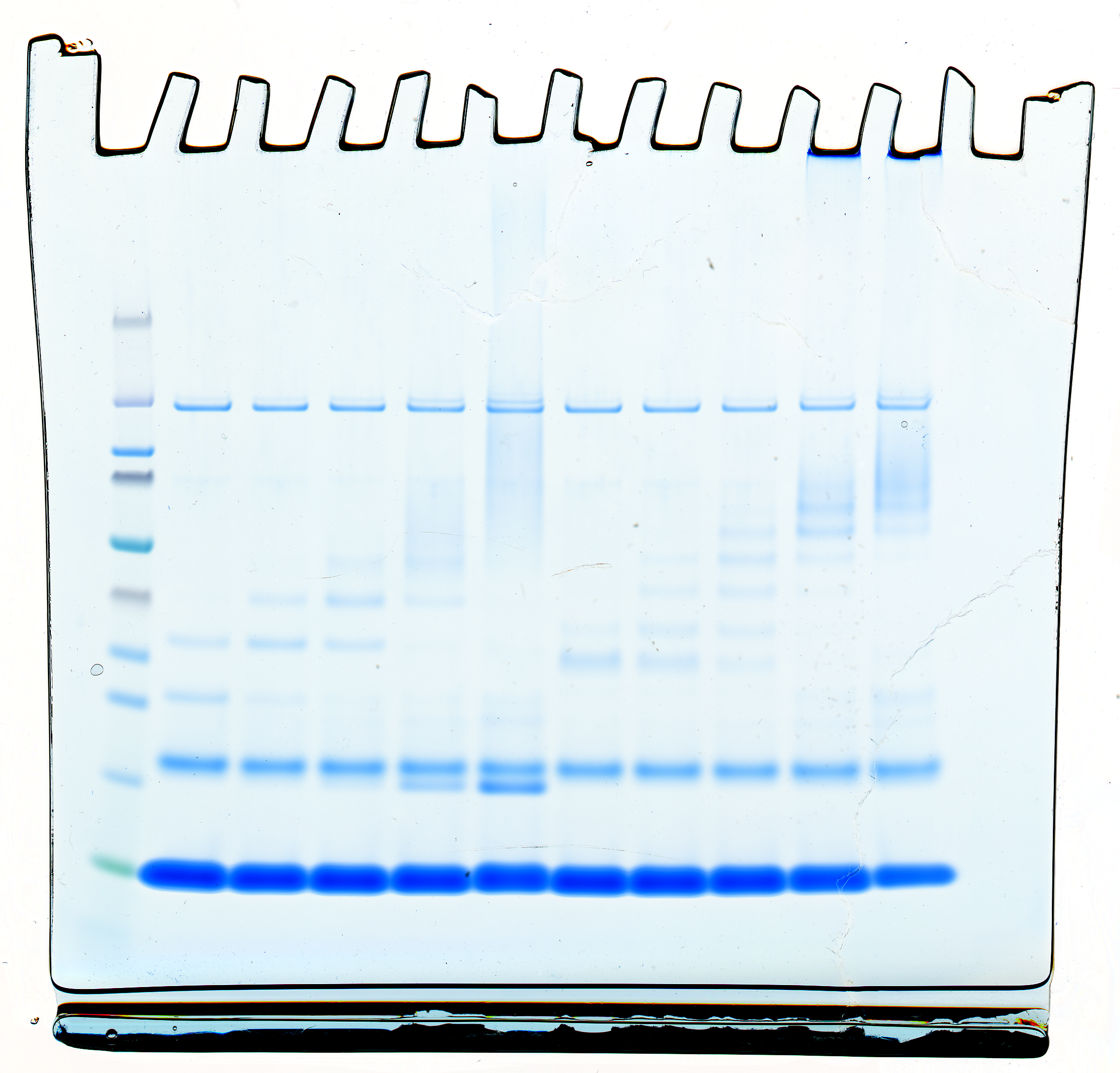

Supplement: Figure 1—source data 3. [file elife-98070-fig1-data3.zip › Figure 1_source data 3/1J.tif]

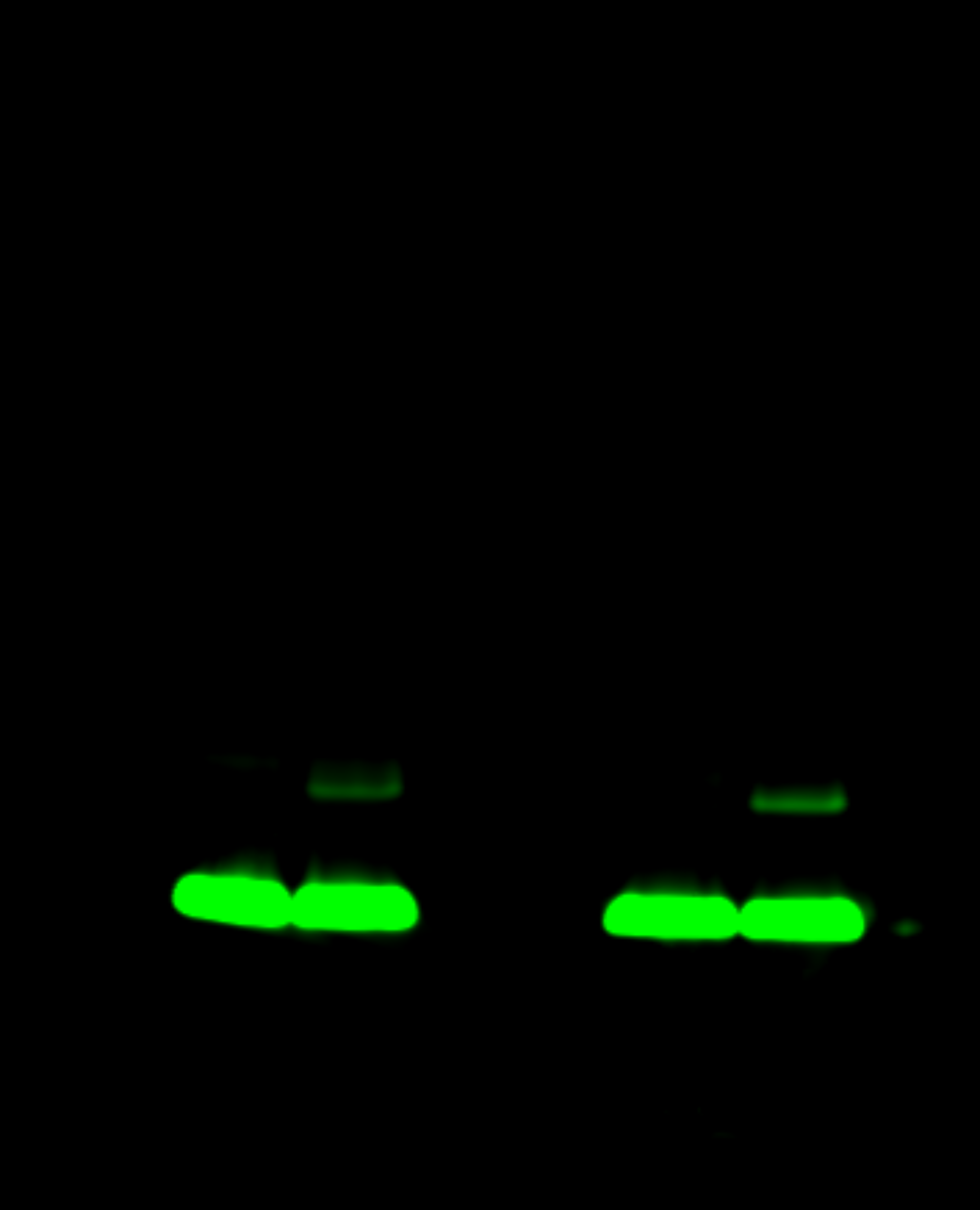

Supplement: Figure 1—source data 3. [file elife-98070-fig1-data3.zip › Figure 1_source data 3/1K.tif]

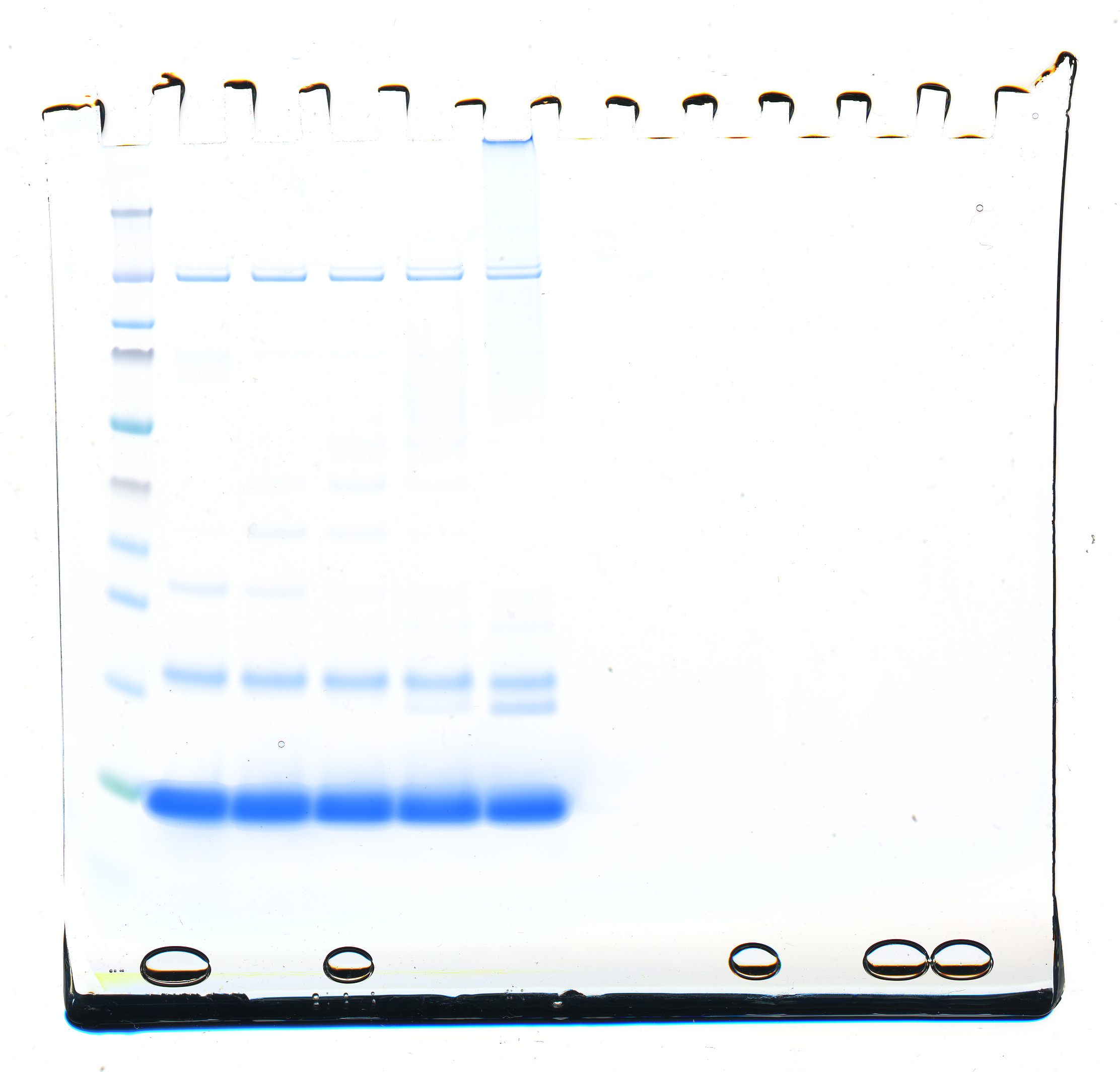

Supplement: Figure 1—source data 3. [file elife-98070-fig1-data3.zip › Figure 1_source data 3/1L.tif]

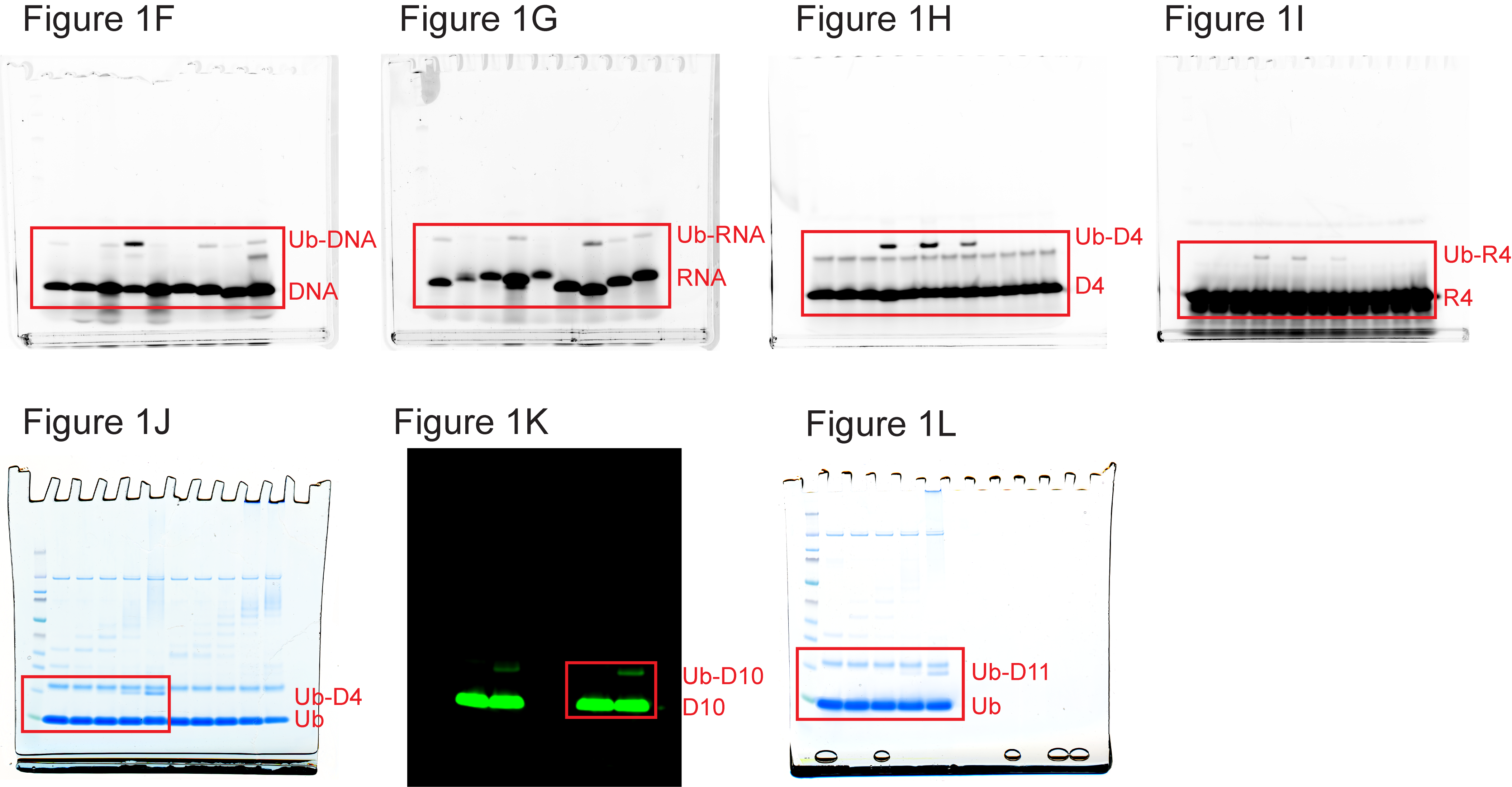

Supplement: Figure 1—source data 4. [file elife-98070-fig1-data4.zip › Figure1_source data 4/Figure1-labelled images.tif]

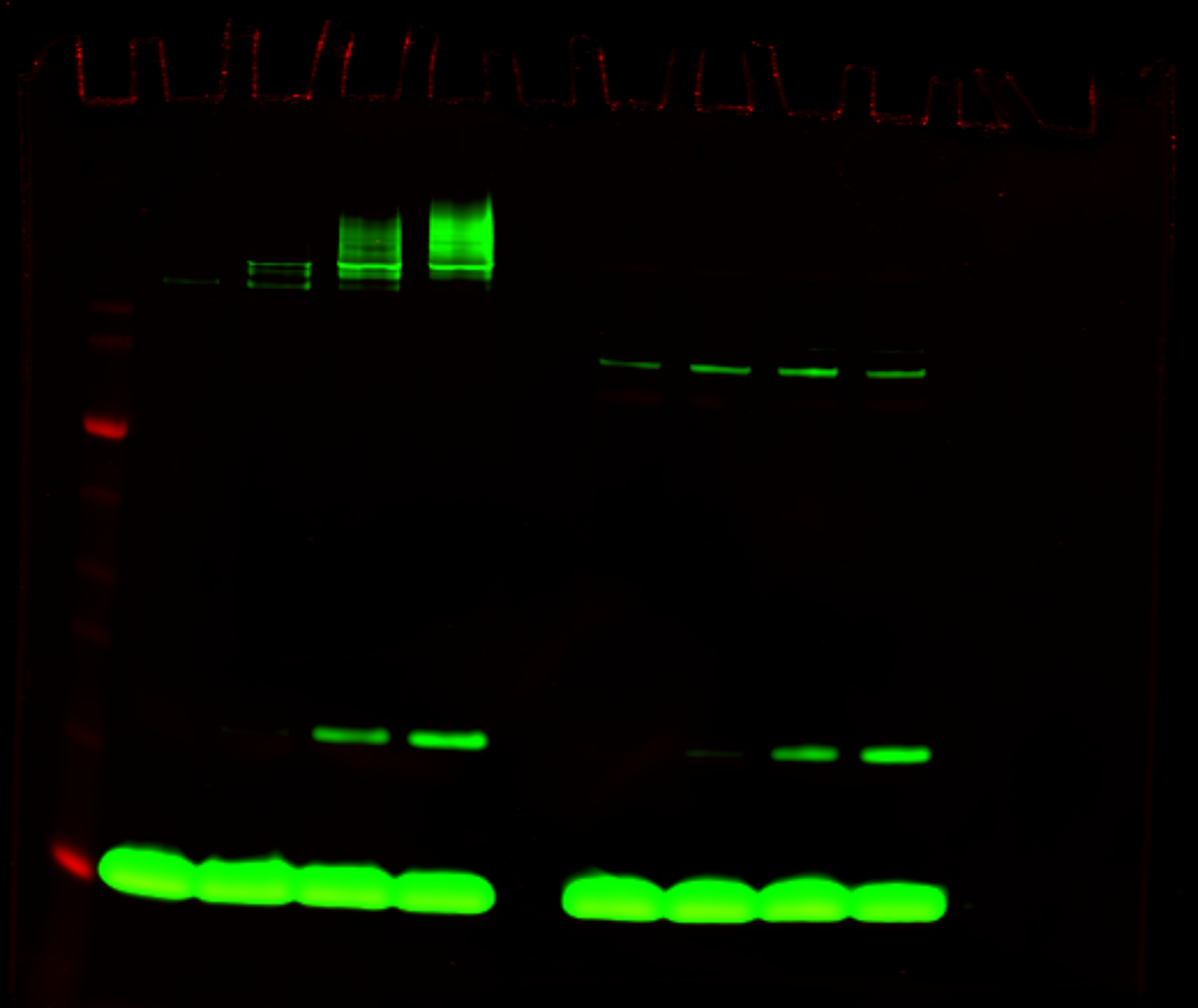

Supplement: Figure 1—figure supplement 1—source data 5. [file elife-98070-fig1-figsupp1-data5.zip › Figure 1_figure supplement 1_source data 5/S1G.tif]

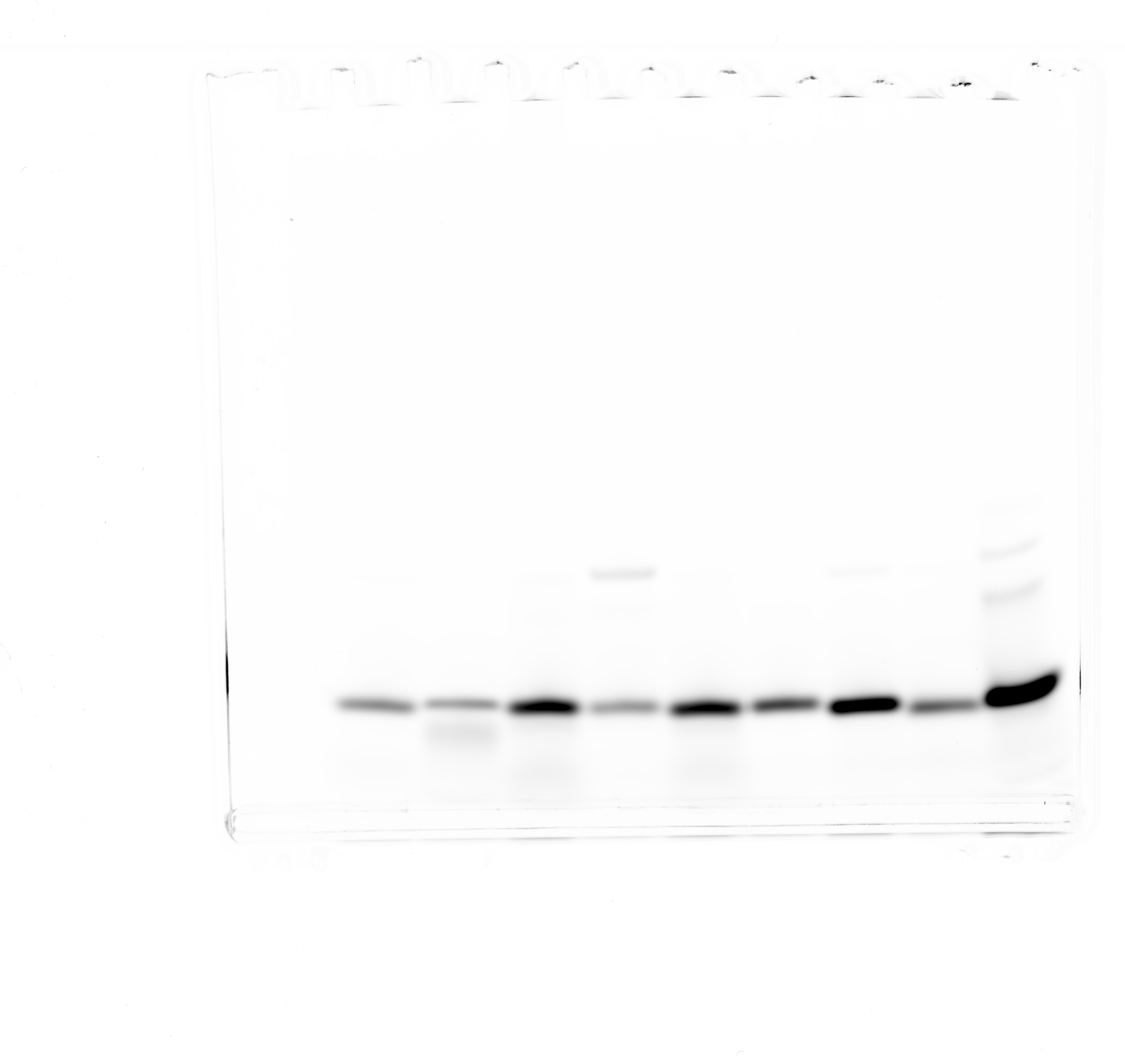

Supplement: Figure 1—figure supplement 1—source data 5. [file elife-98070-fig1-figsupp1-data5.zip › Figure 1_figure supplement 1_source data 5/S1K.tif]

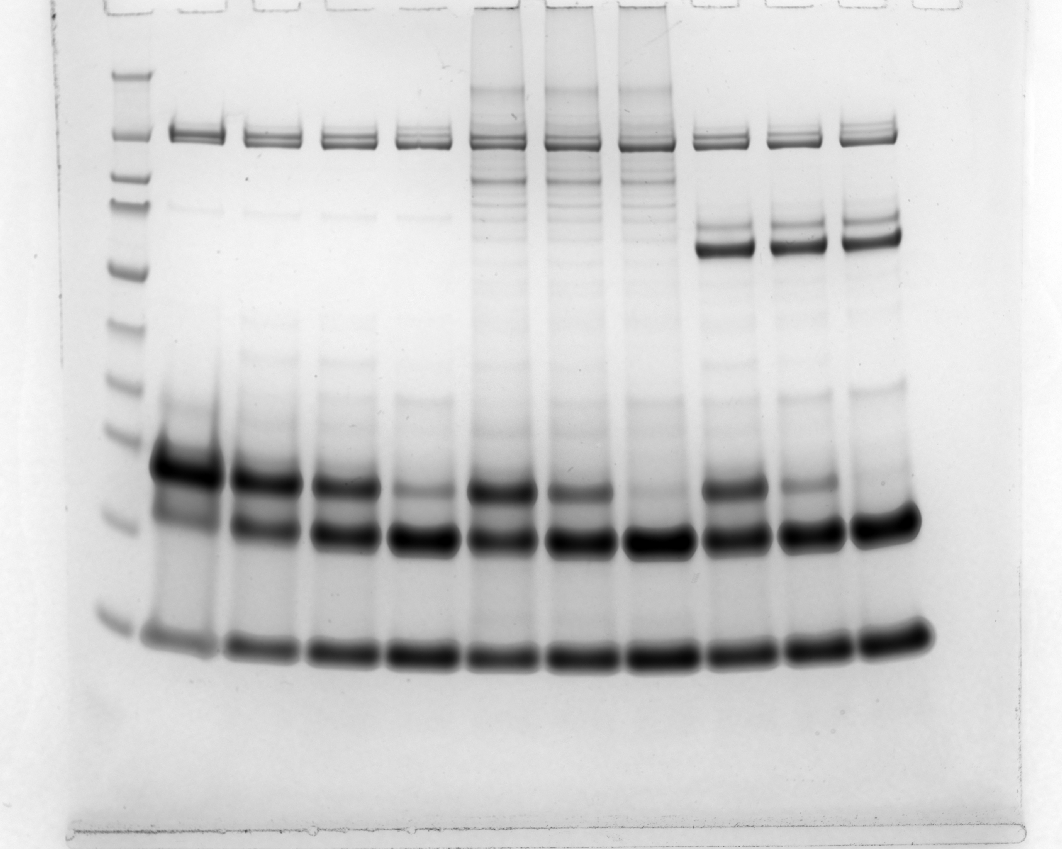

Supplement: Figure 1—figure supplement 1—source data 5. [file elife-98070-fig1-figsupp1-data5.zip › Figure 1_figure supplement 1_source data 5/S1H.tif]

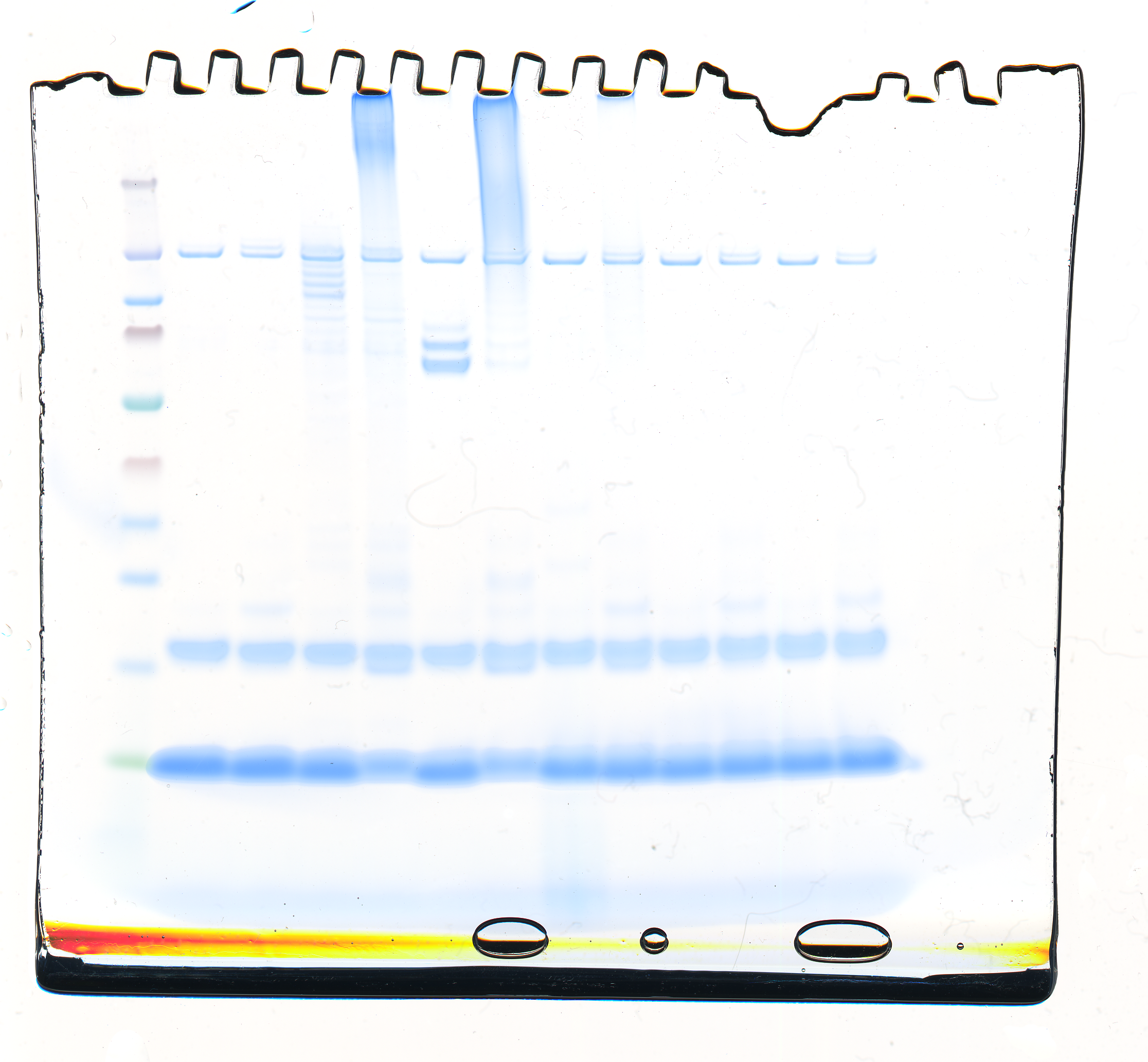

Supplement: Figure 1—figure supplement 1—source data 5. [file elife-98070-fig1-figsupp1-data5.zip › Figure 1_figure supplement 1_source data 5/S1I.tif]

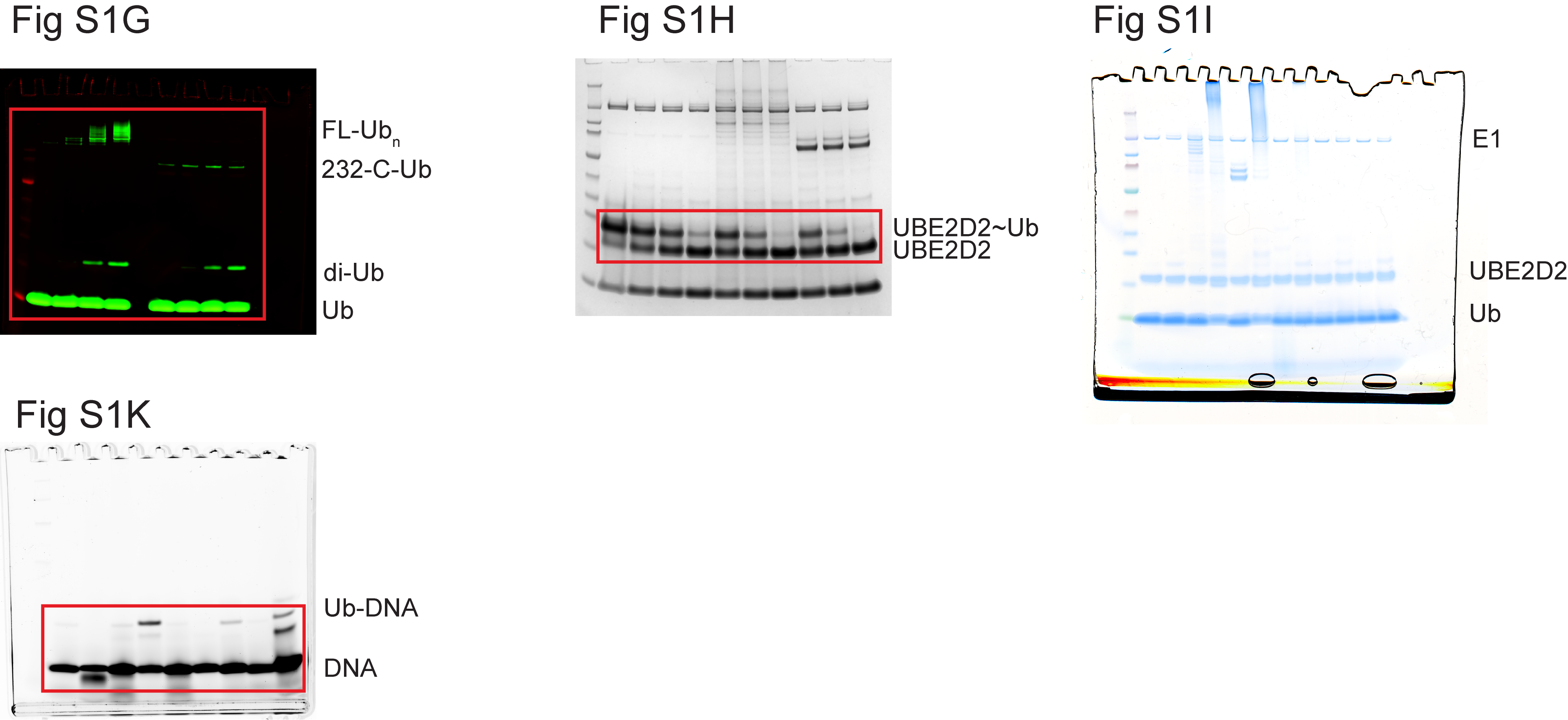

Supplement: Figure 1—figure supplement 1—source data 6. [file elife-98070-fig1-figsupp1-data6.zip › Figure 1_figure supplement 1_source data 6/Figure1_figure supplement 1_labelled images.tif]

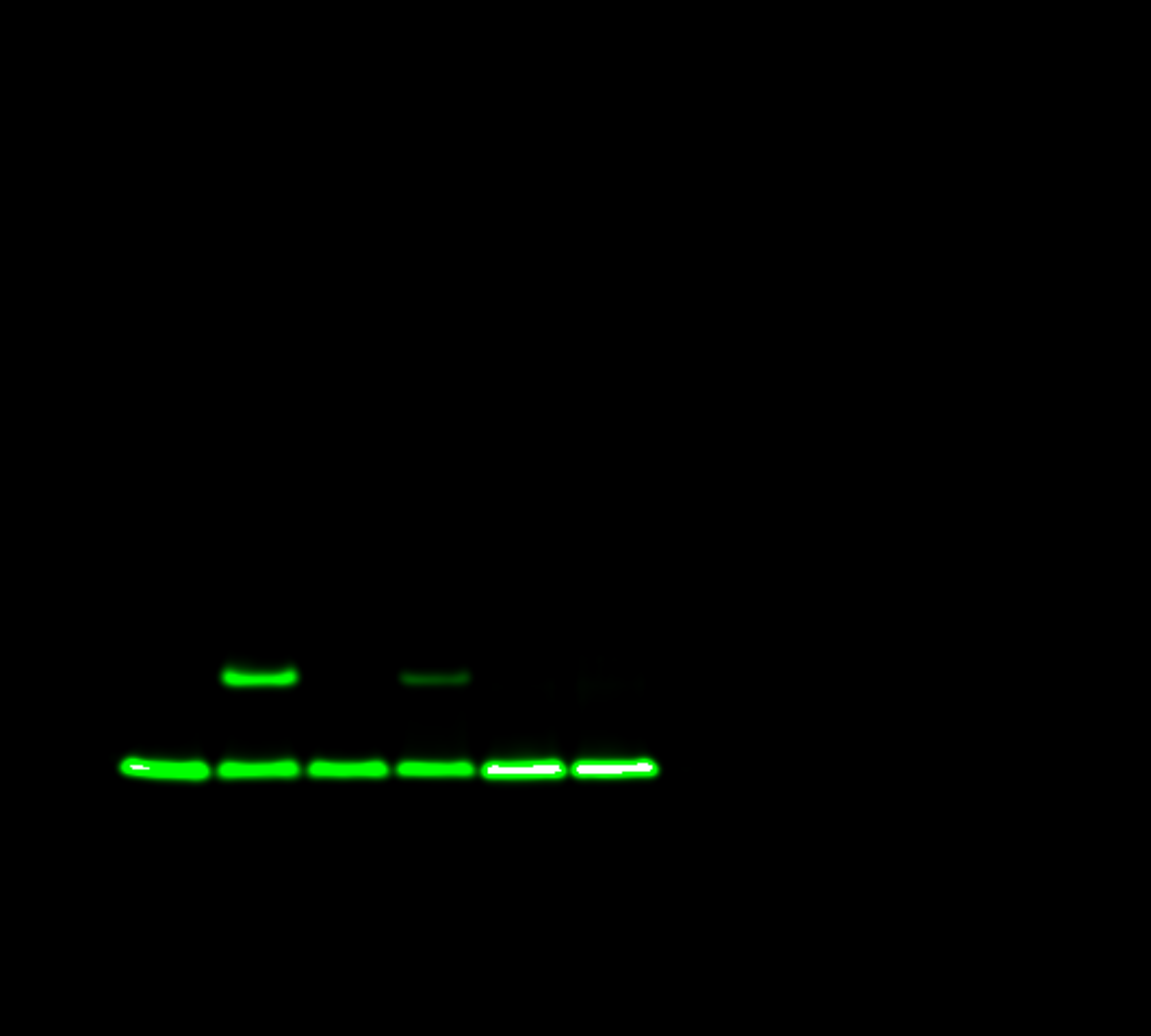

Supplement: Figure 2—source data 1. [file elife-98070-fig2-data1.zip › Figure 2_source data 1/2I.tif]

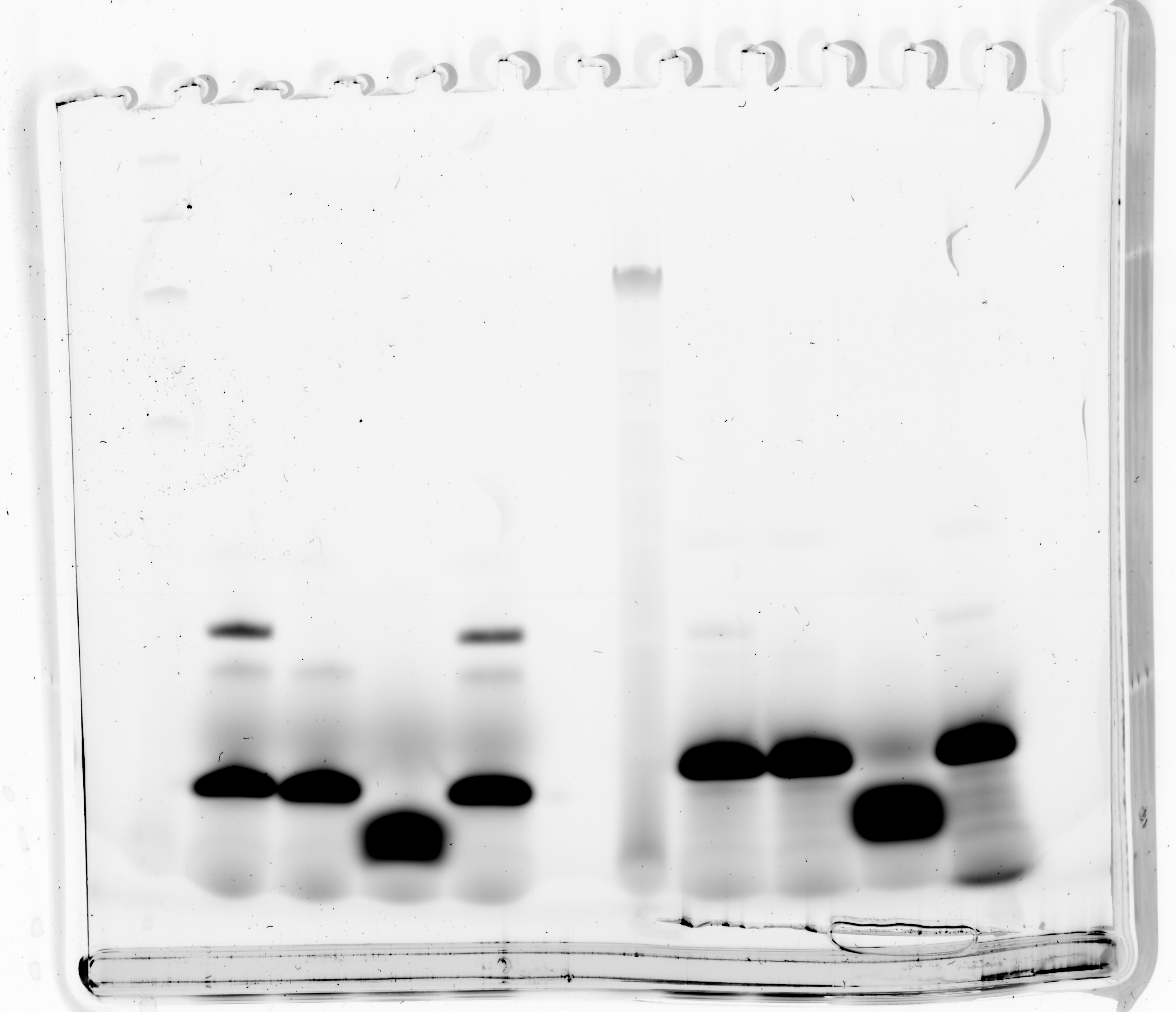

Supplement: Figure 2—source data 1. [file elife-98070-fig2-data1.zip › Figure 2_source data 1/2C_D.tif]

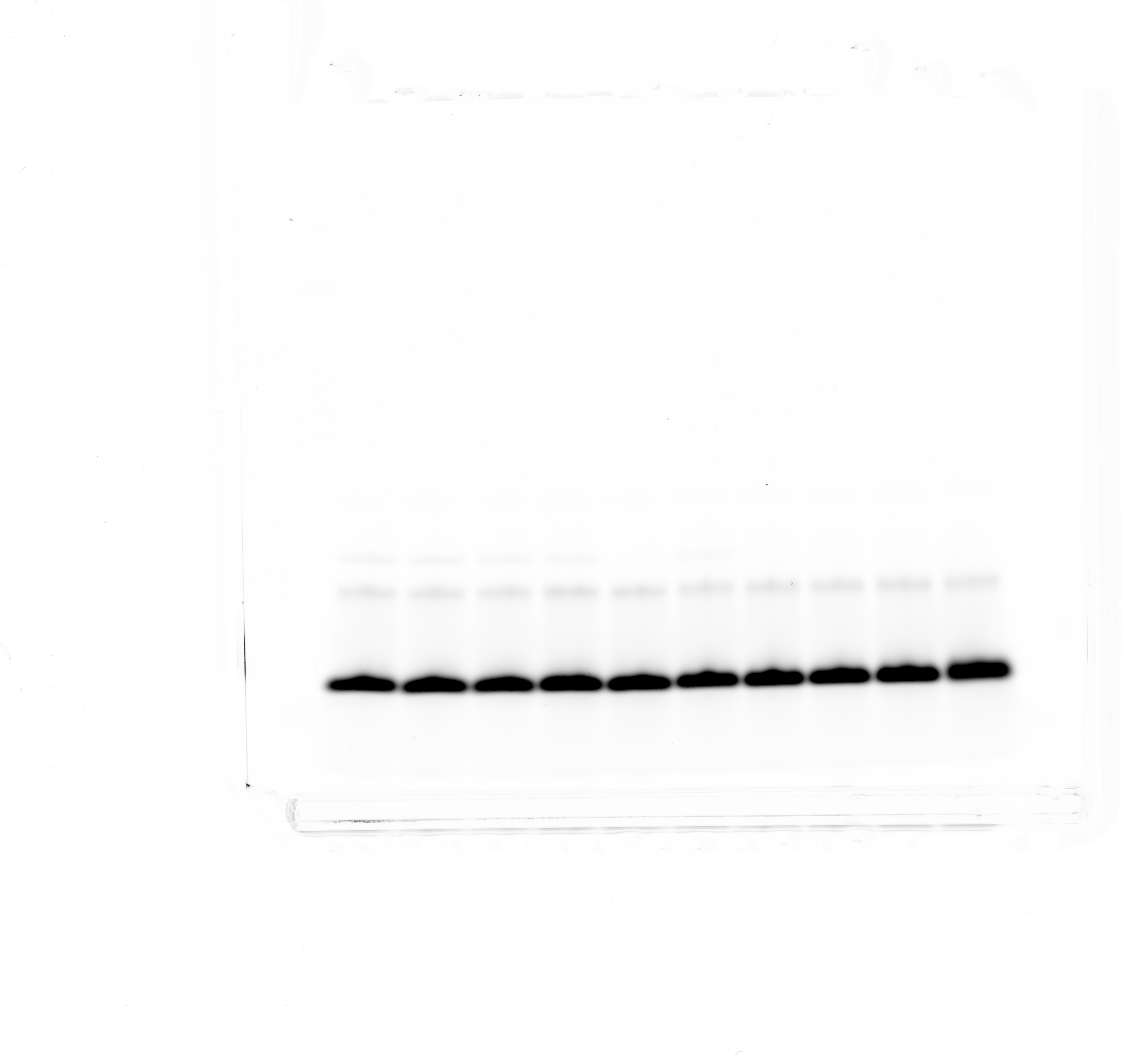

Supplement: Figure 2—source data 1. [file elife-98070-fig2-data1.zip › Figure 2_source data 1/2H.tif]

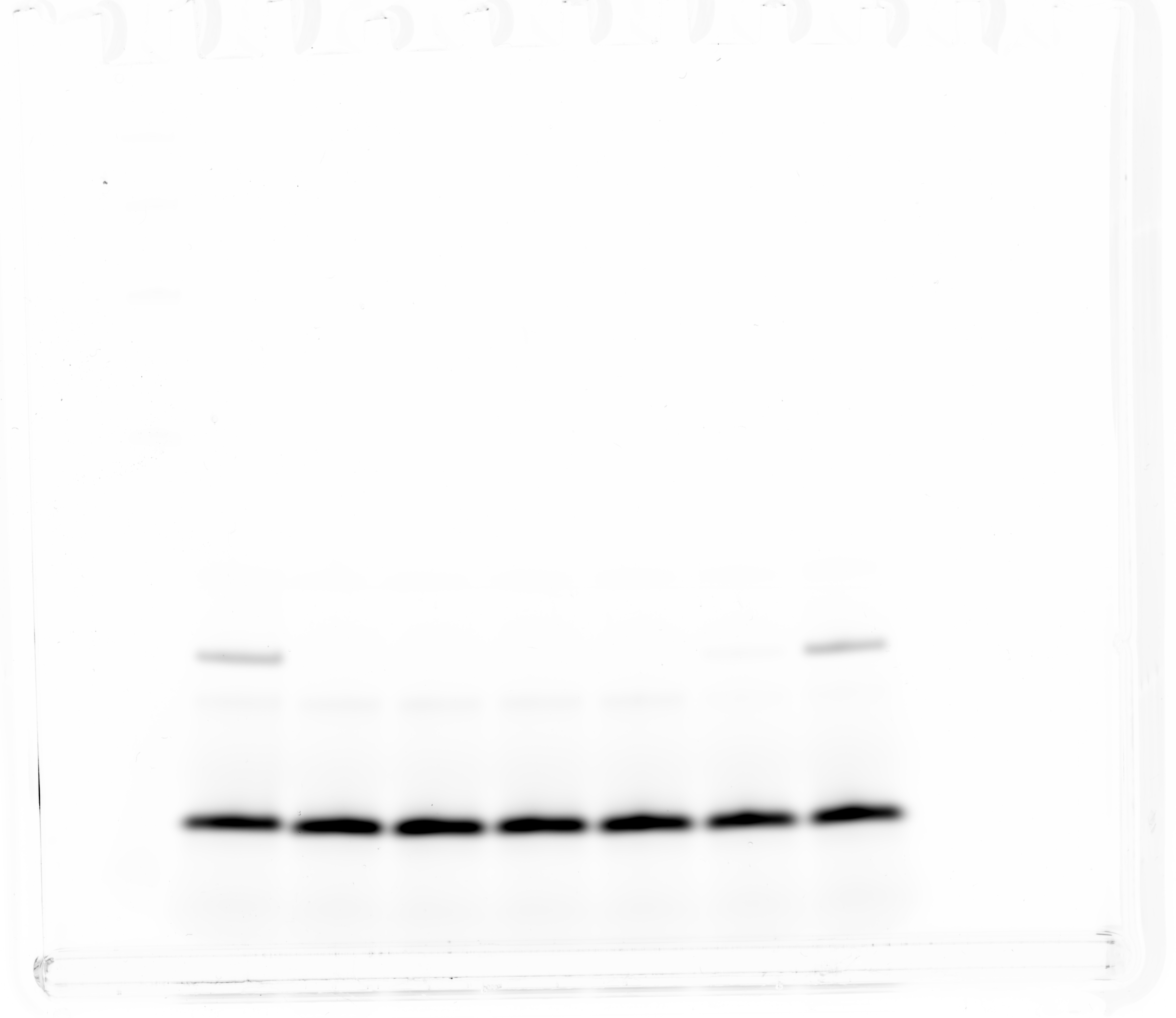

Supplement: Figure 2—source data 1. [file elife-98070-fig2-data1.zip › Figure 2_source data 1/2A.tif]

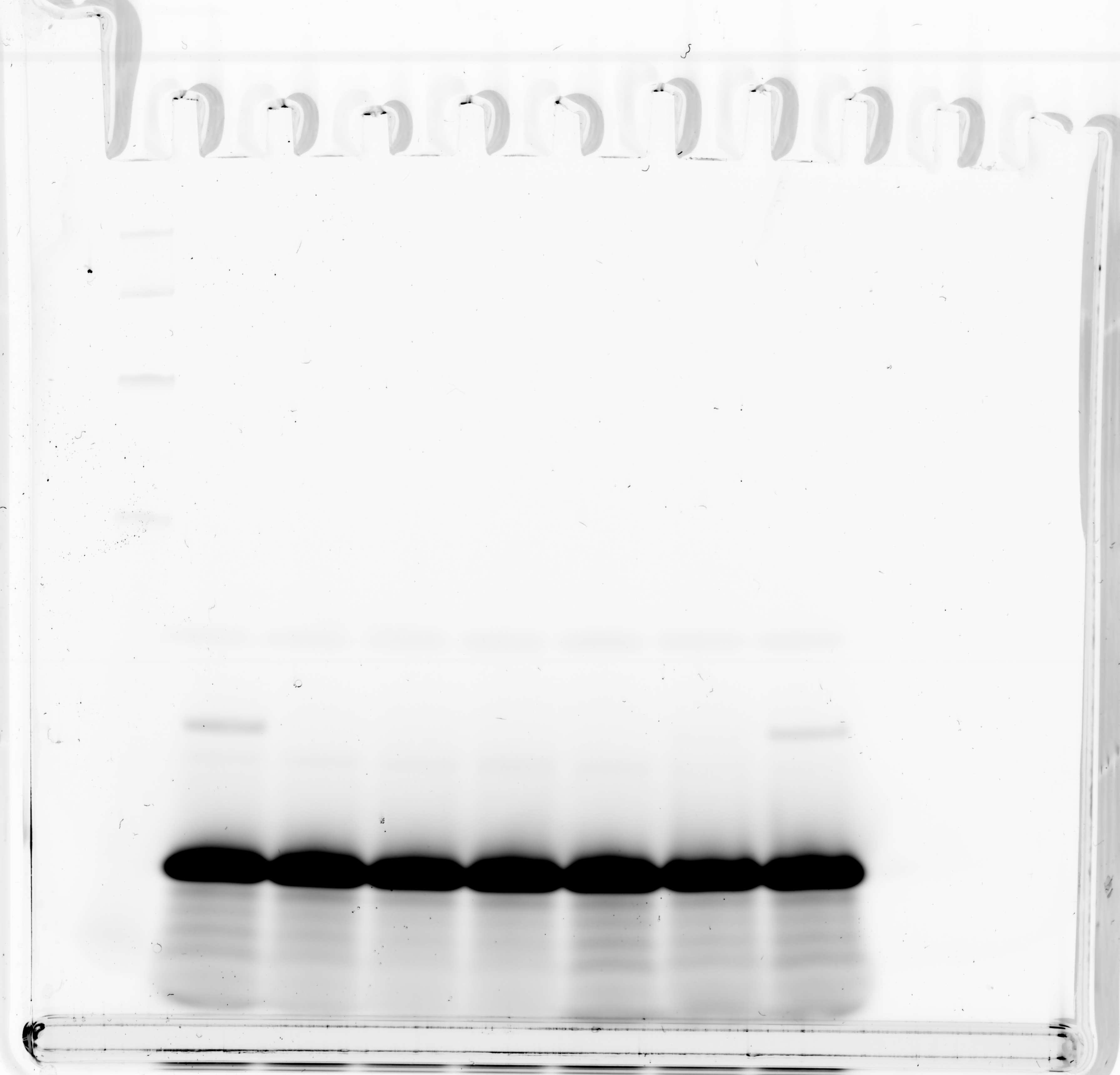

Supplement: Figure 2—source data 1. [file elife-98070-fig2-data1.zip › Figure 2_source data 1/2B.tif]

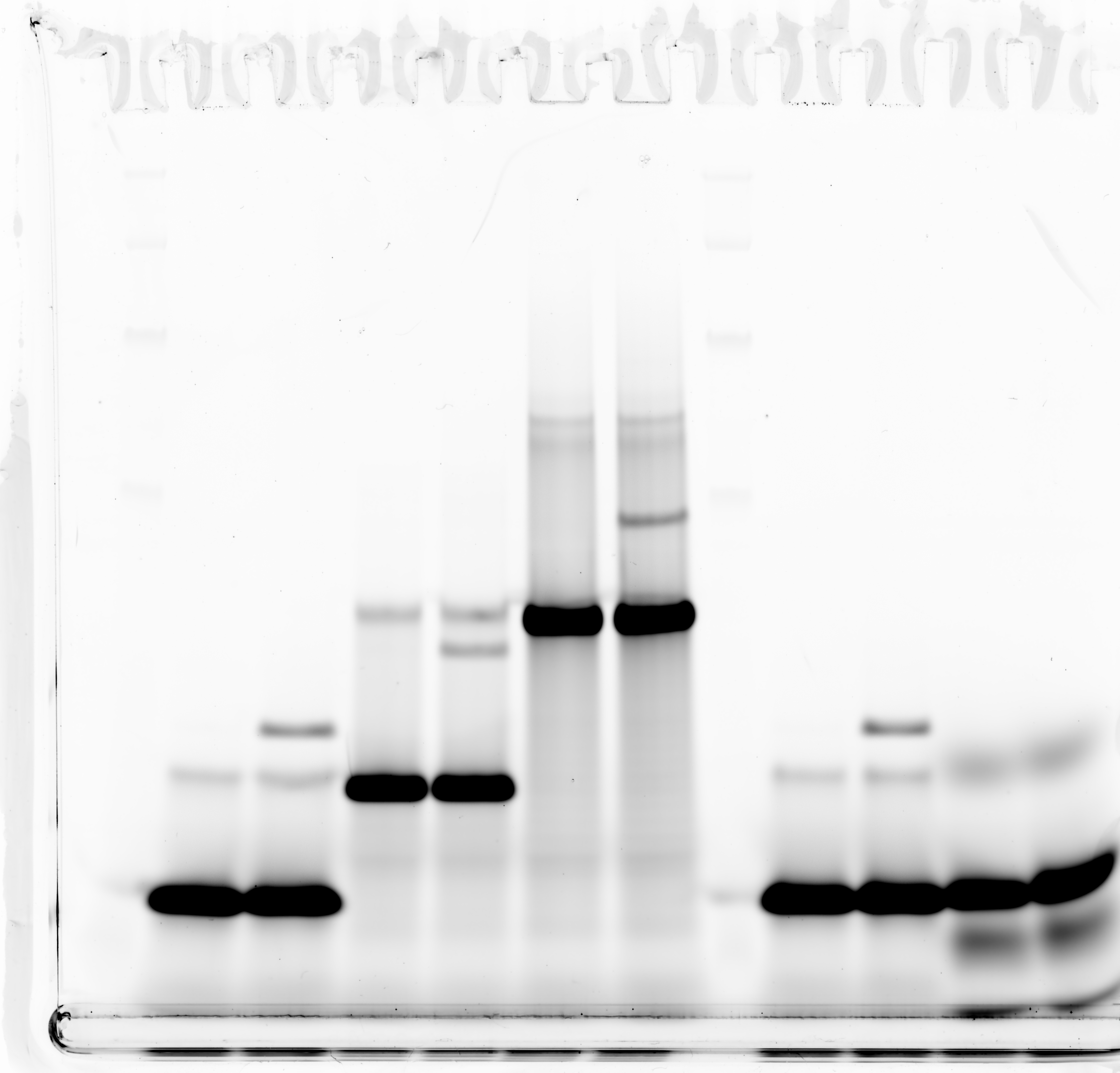

Supplement: Figure 2—source data 1. [file elife-98070-fig2-data1.zip › Figure 2_source data 1/2F.tif]

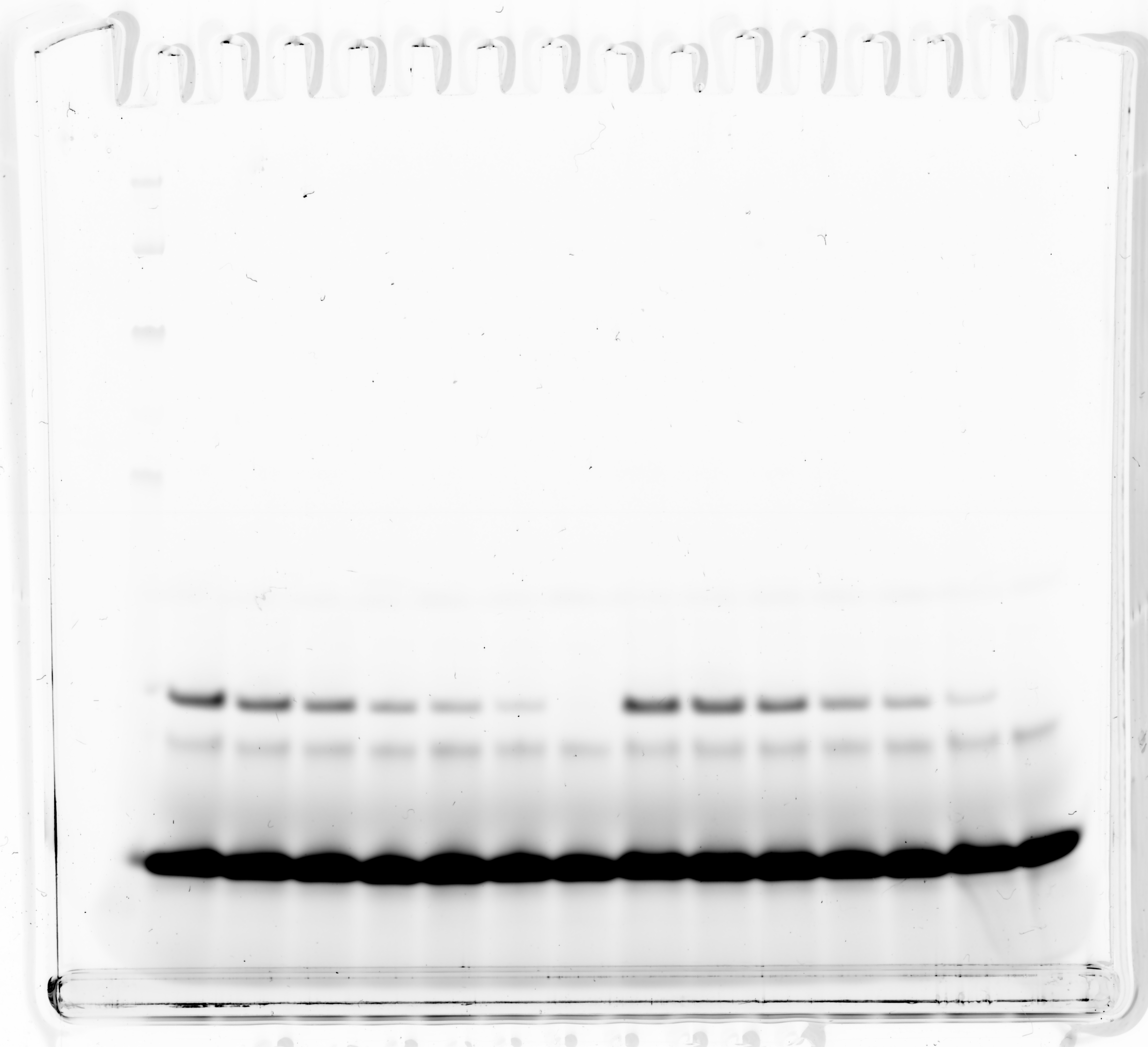

Supplement: Figure 2—source data 1. [file elife-98070-fig2-data1.zip › Figure 2_source data 1/2G.tif]

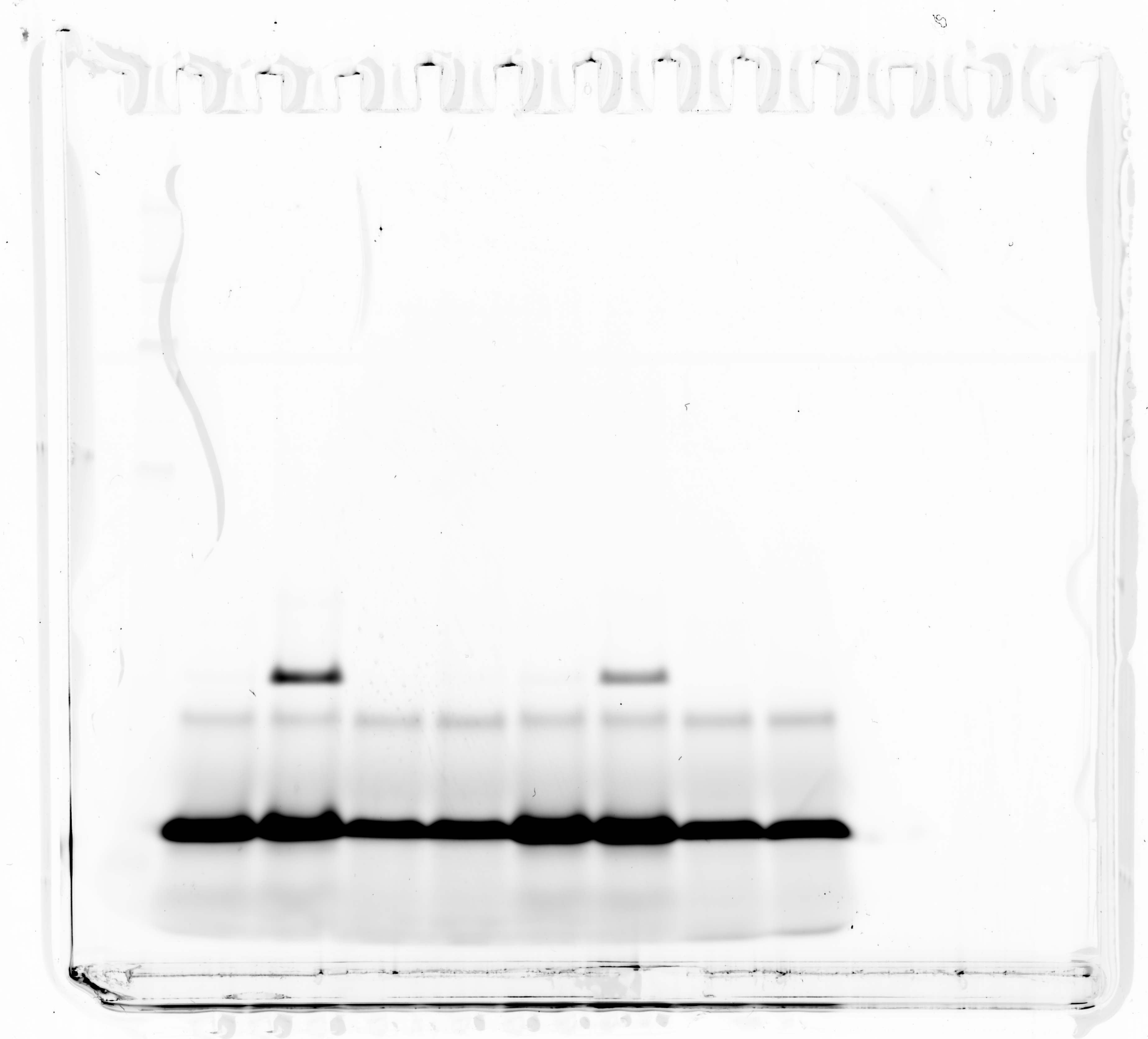

Supplement: Figure 2—source data 1. [file elife-98070-fig2-data1.zip › Figure 2_source data 1/2E.tif]

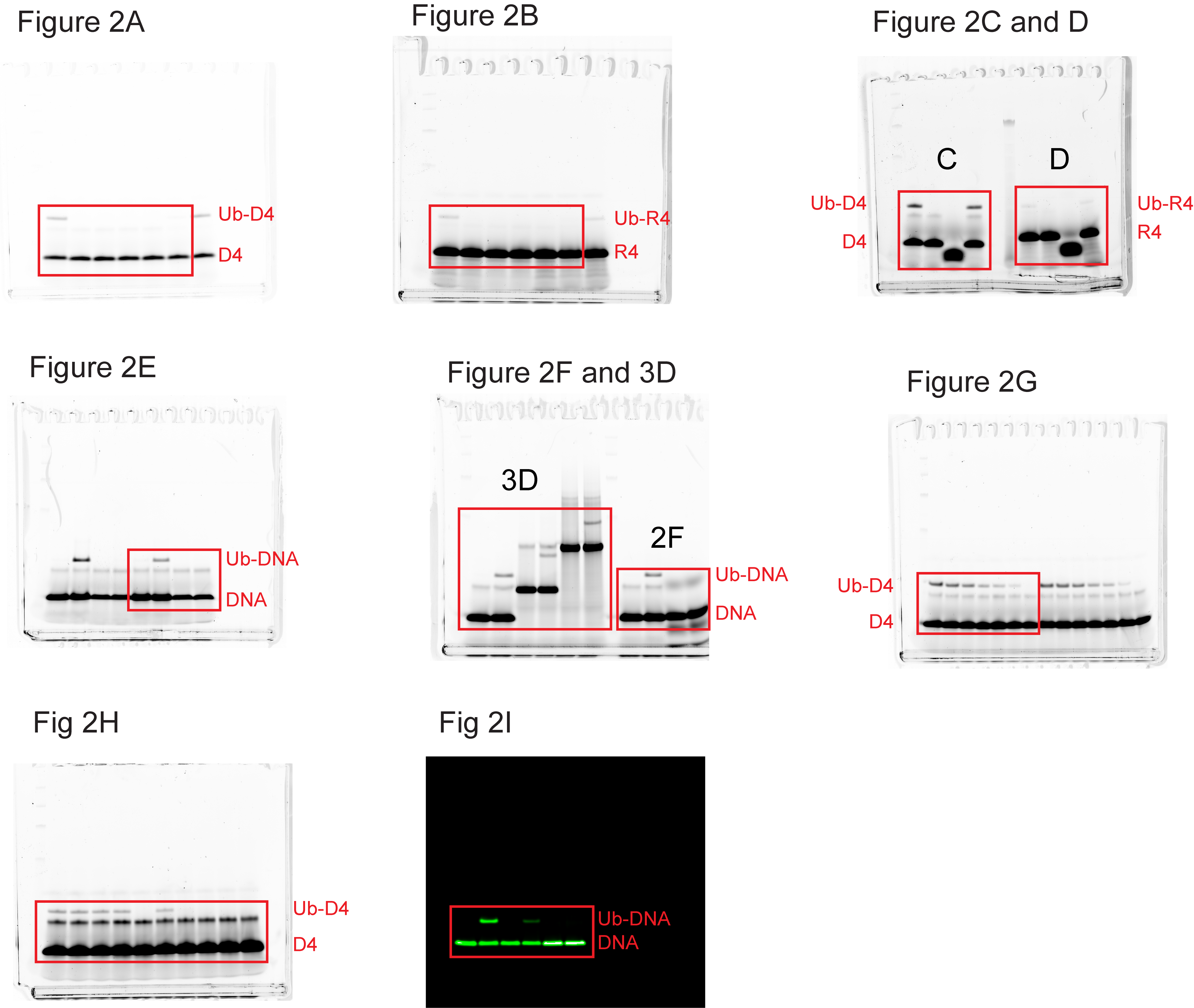

Supplement: Figure 2—source data 2. [file elife-98070-fig2-data2.zip › Figure 2_source data 2/Figure 2_labelled images.tif]

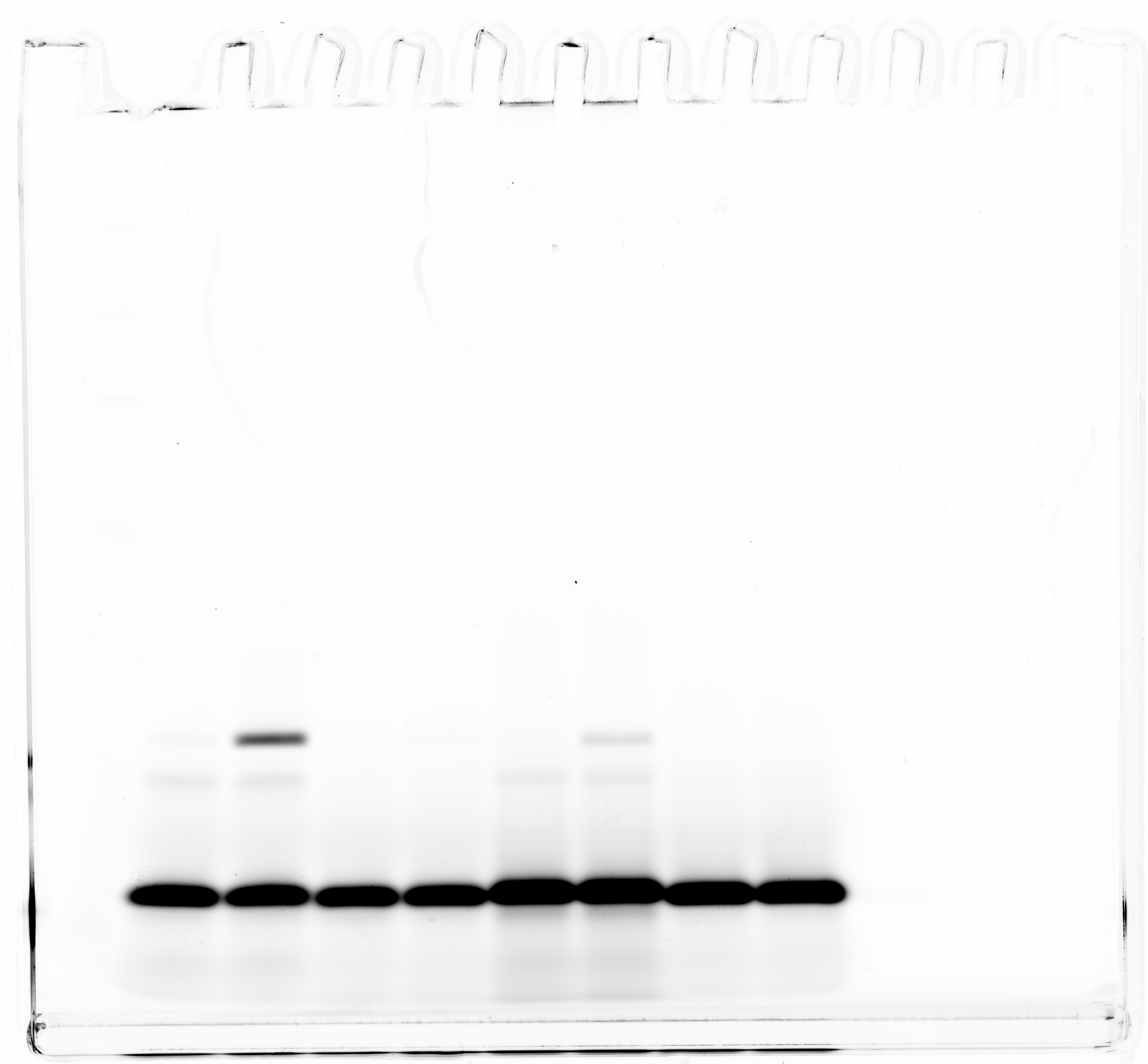

Supplement: Figure 3—source data 1. [file elife-98070-fig3-data1.zip › Figure 3_source data 1/3A.tif]

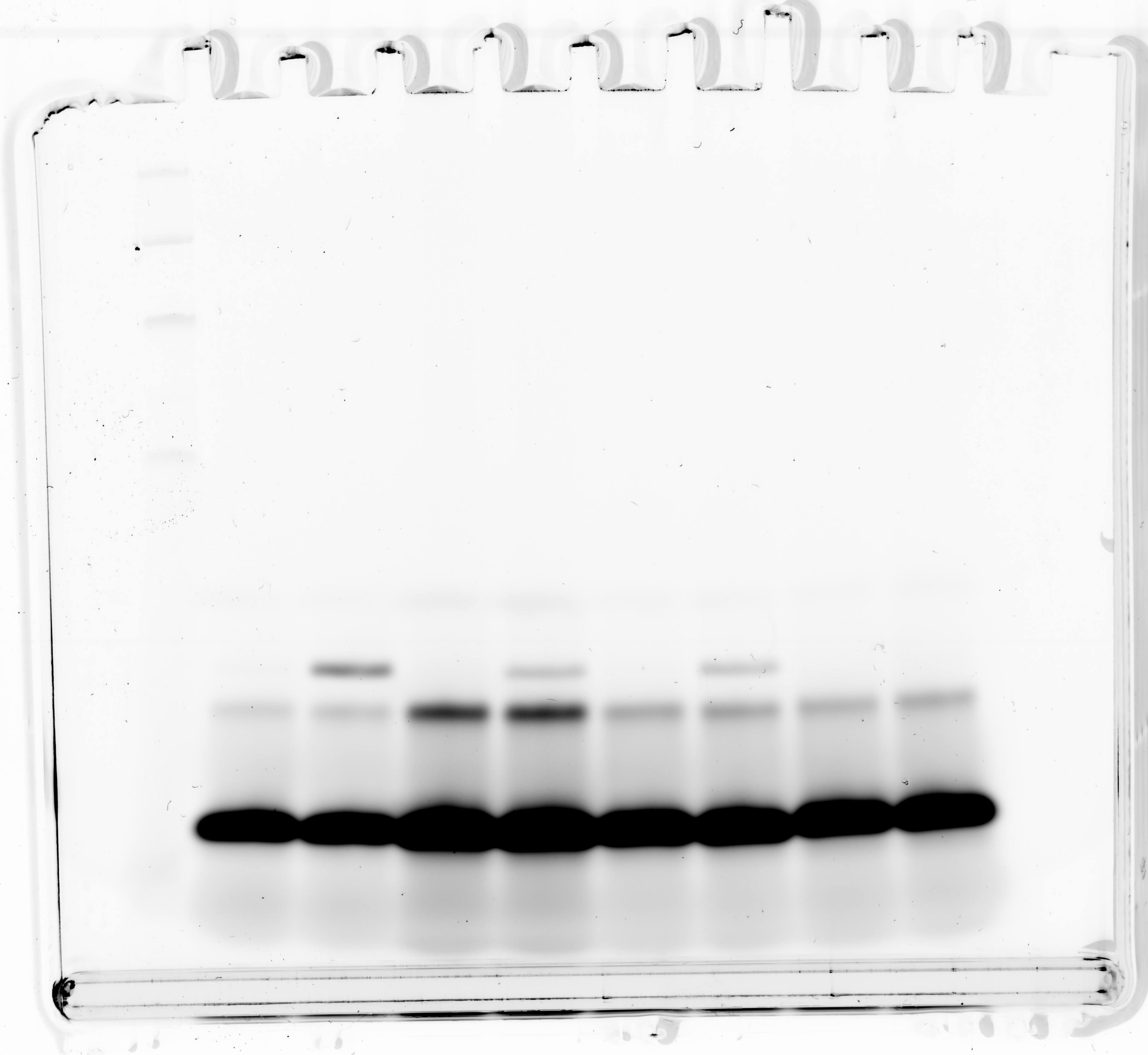

Supplement: Figure 3—source data 1. [file elife-98070-fig3-data1.zip › Figure 3_source data 1/3B.tif]

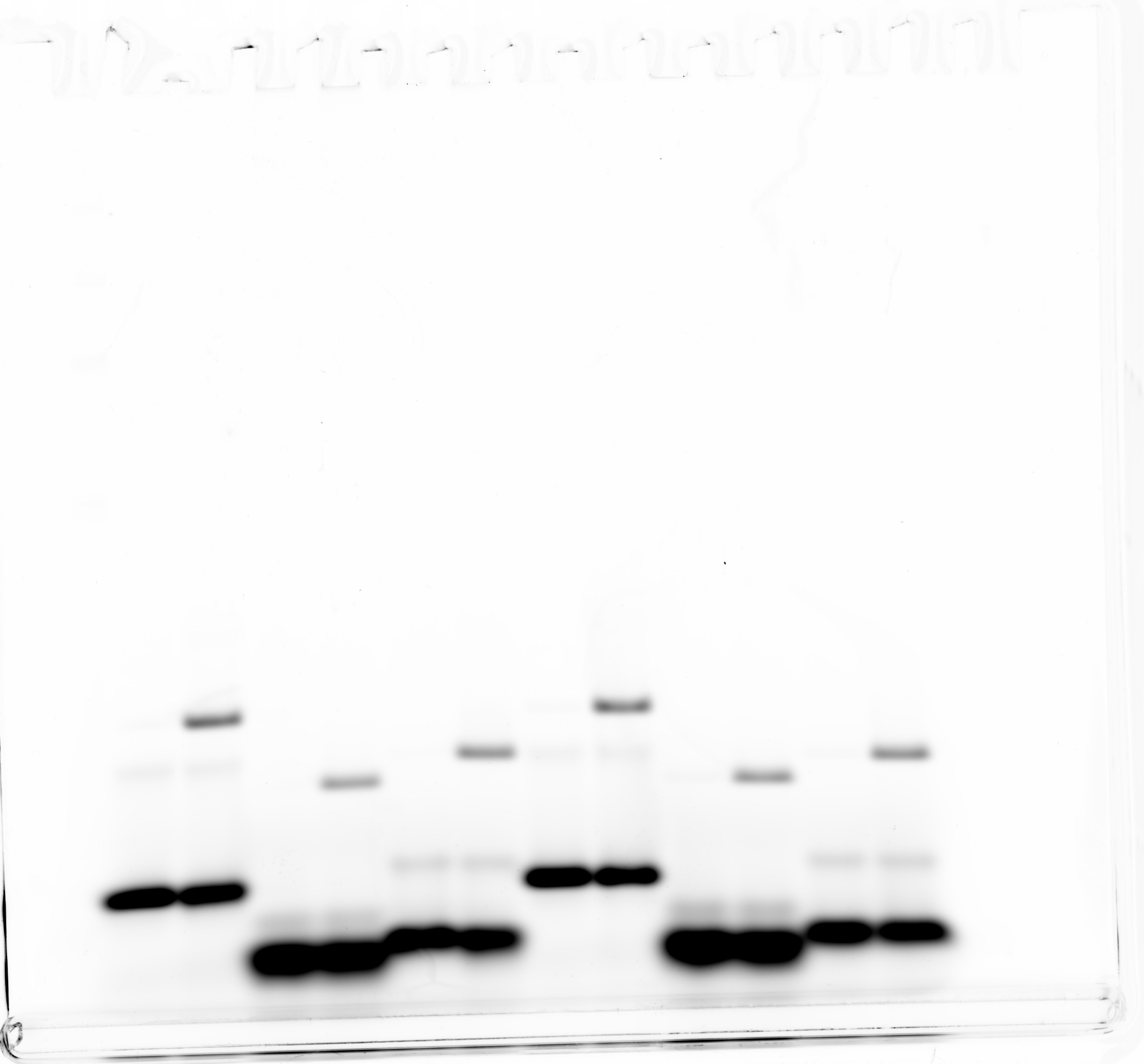

Supplement: Figure 3—source data 1. [file elife-98070-fig3-data1.zip › Figure 3_source data 1/3C.tif]

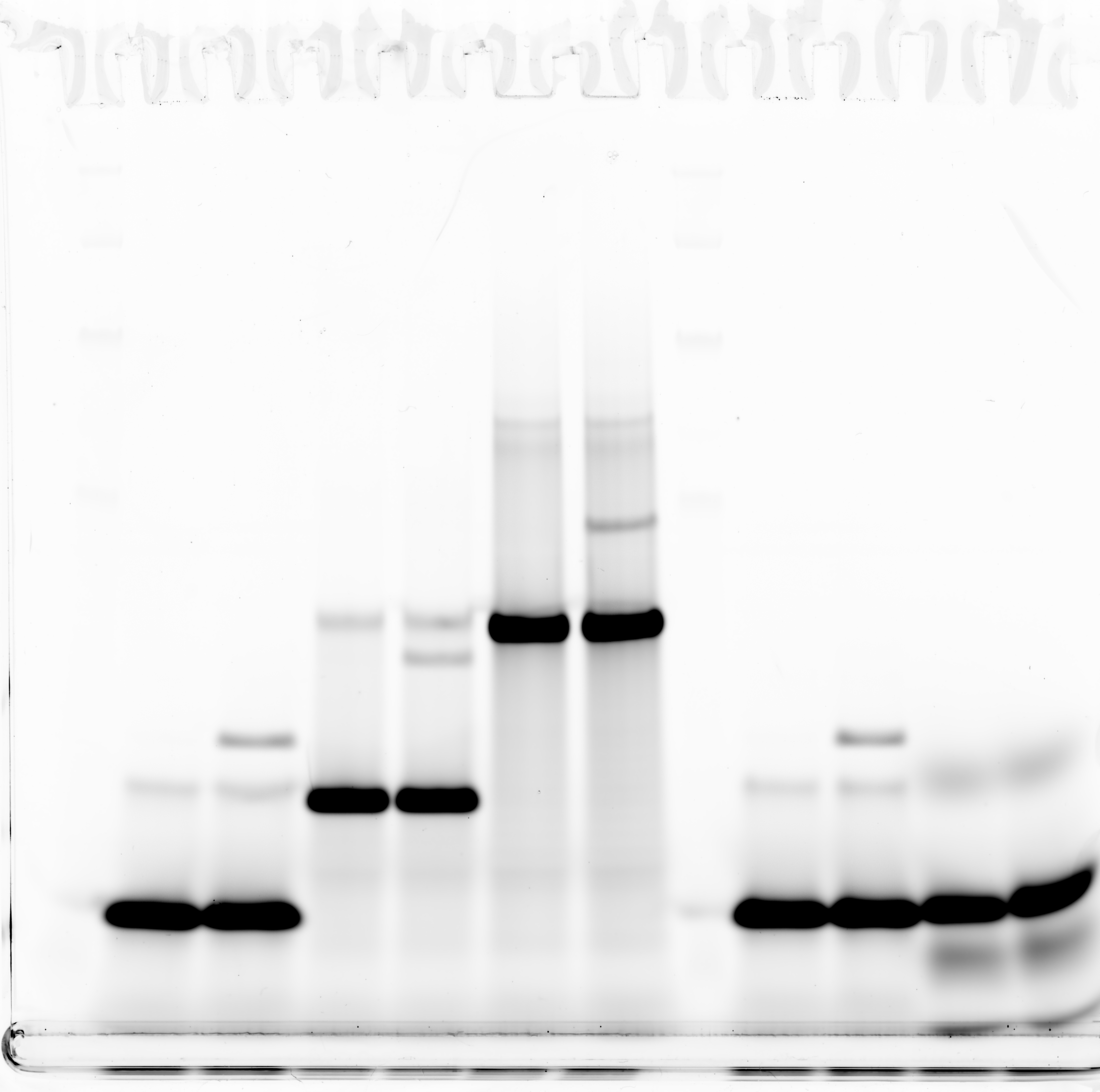

Supplement: Figure 3—source data 1. [file elife-98070-fig3-data1.zip › Figure 3_source data 1/3D.tif]

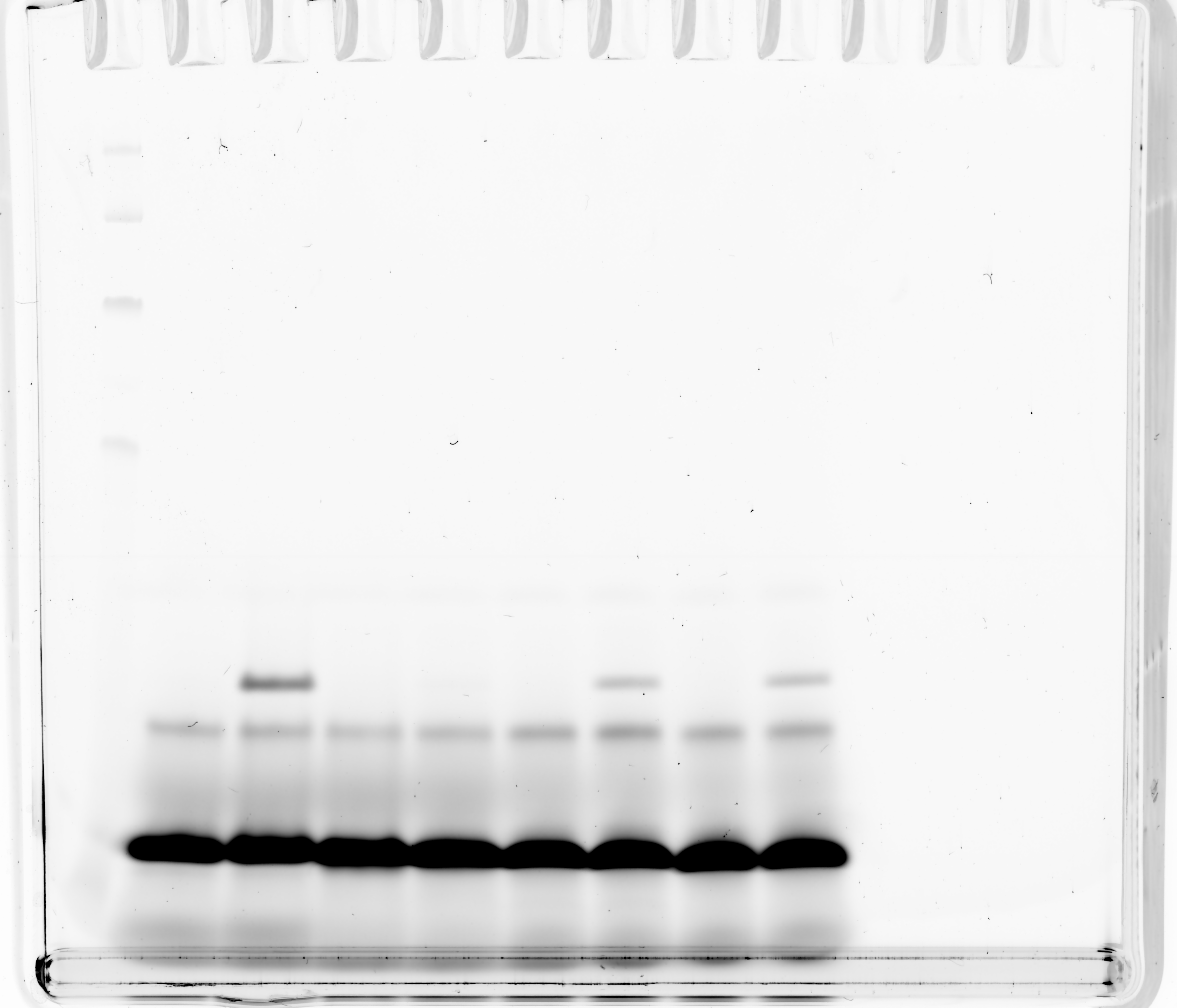

Supplement: Figure 3—source data 1. [file elife-98070-fig3-data1.zip › Figure 3_source data 1/3E.tif]

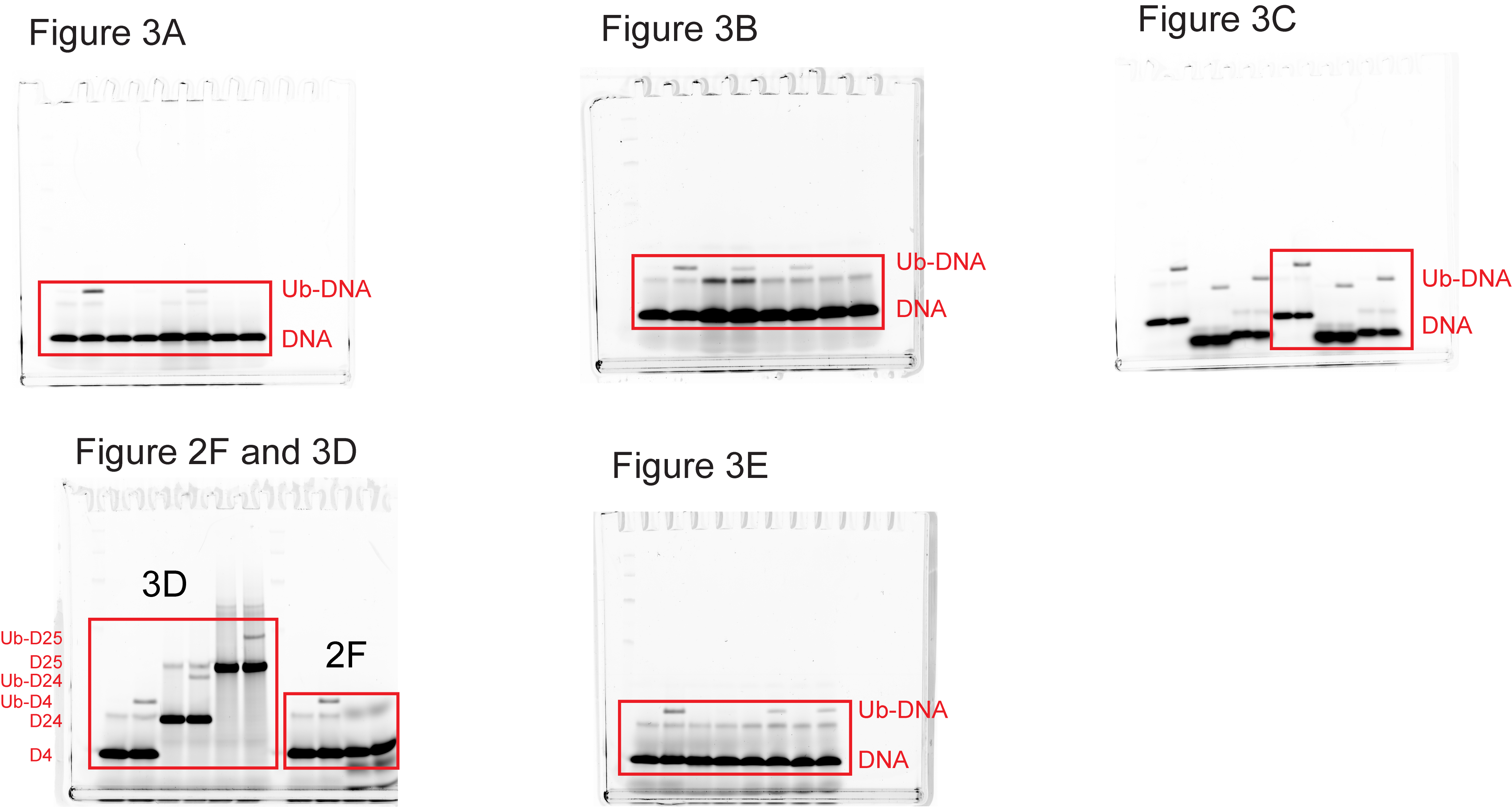

Supplement: Figure 3—source data 2. [file elife-98070-fig3-data2.zip › Figure 3_source data 2/figure 3_labelled images.tif]

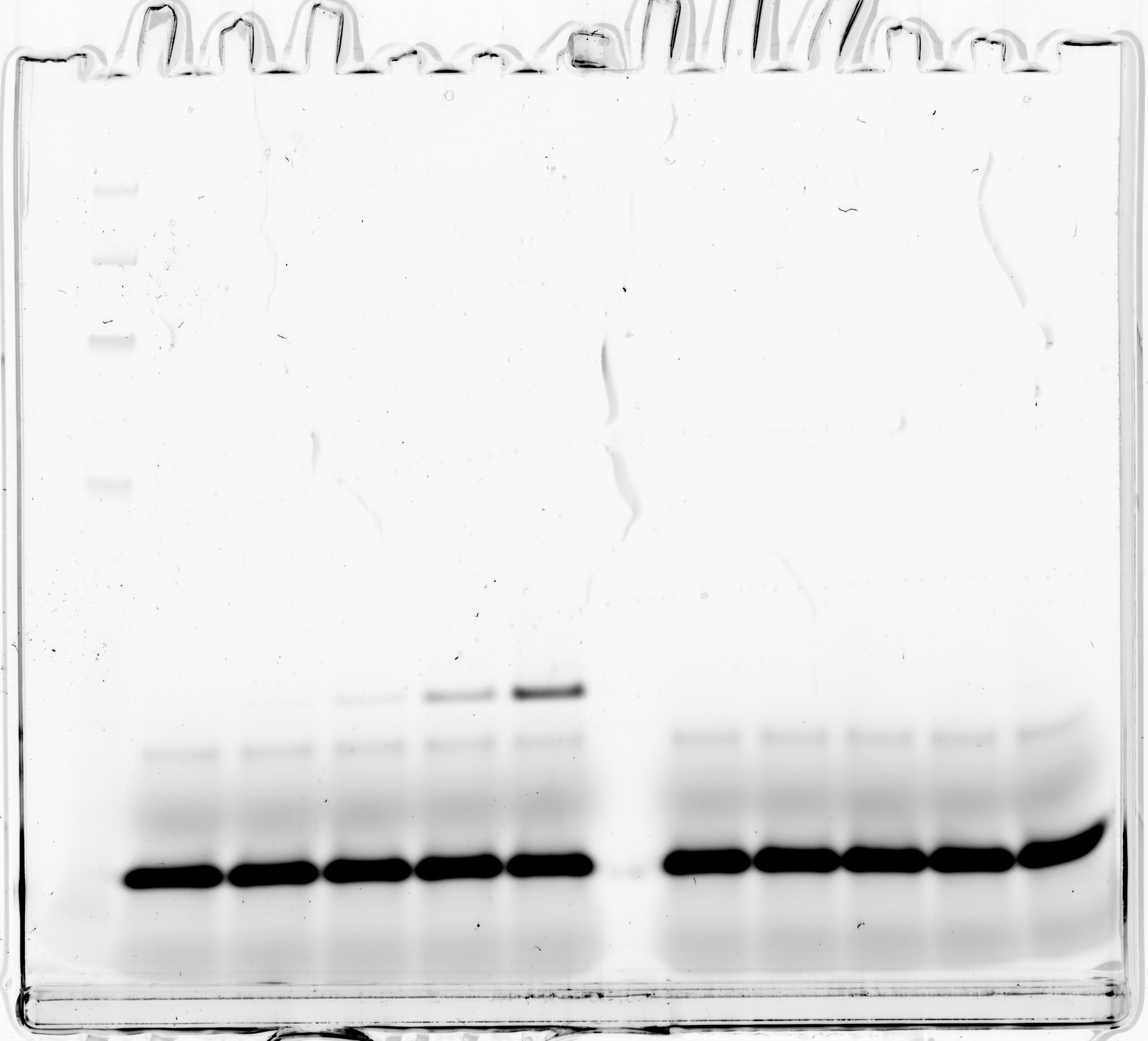

Supplement: Figure 4—source data 1. [file elife-98070-fig4-data1.zip › Figure 4_source data 1/4D.tif]

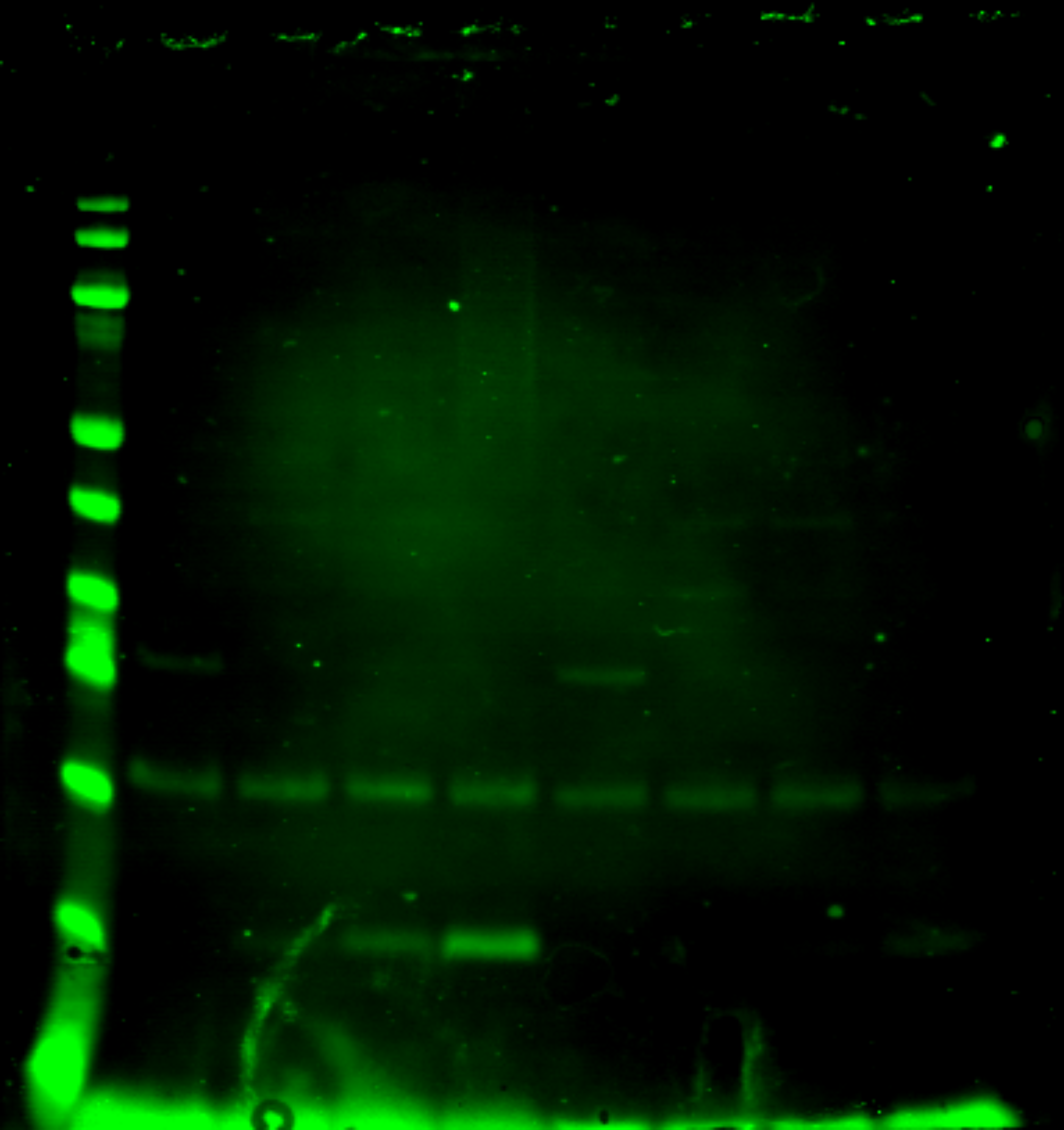

Supplement: Figure 4—source data 1. [file elife-98070-fig4-data1.zip › Figure 4_source data 1/4E.tif]

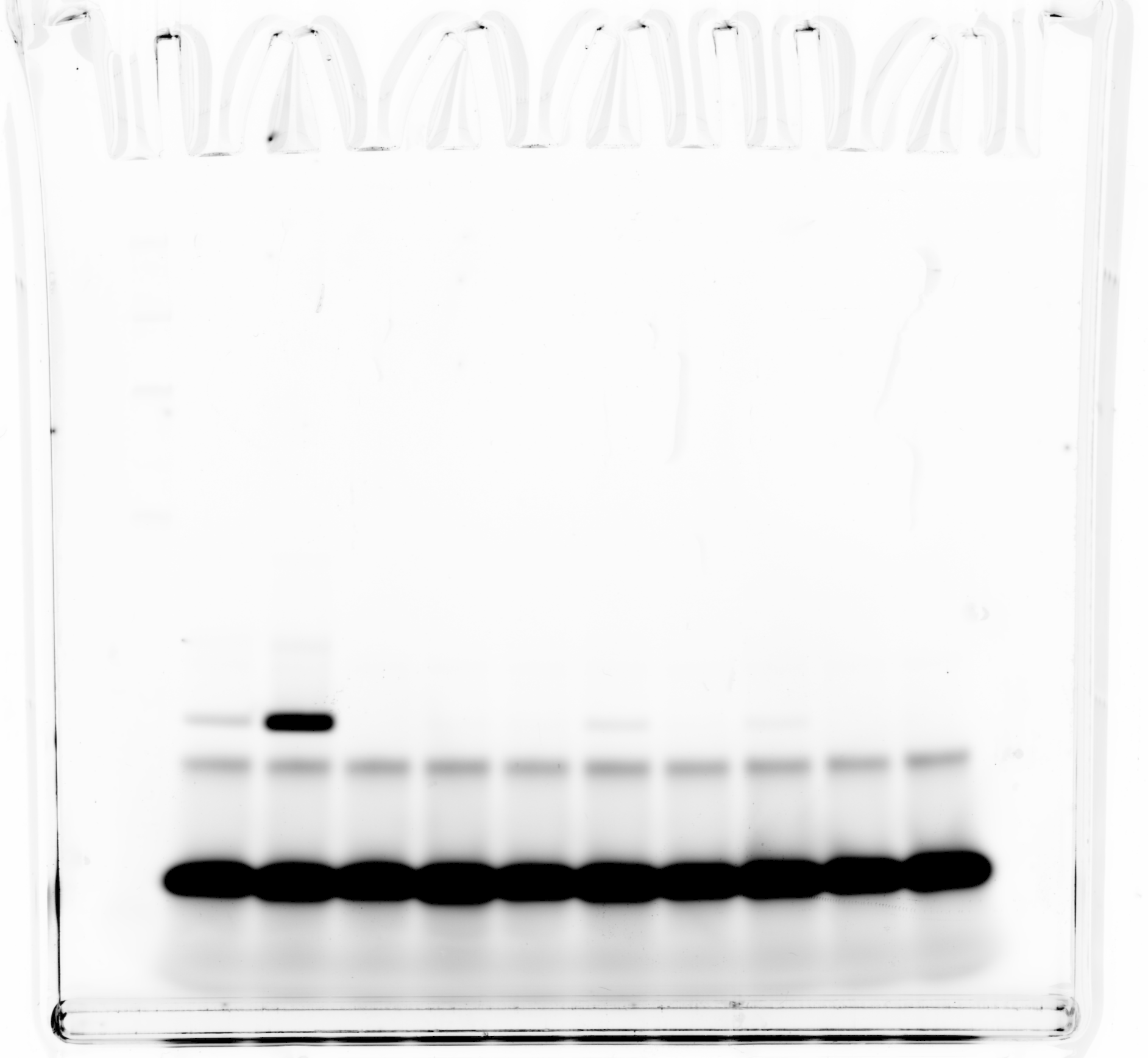

Supplement: Figure 4—source data 1. [file elife-98070-fig4-data1.zip › Figure 4_source data 1/4I.tif]

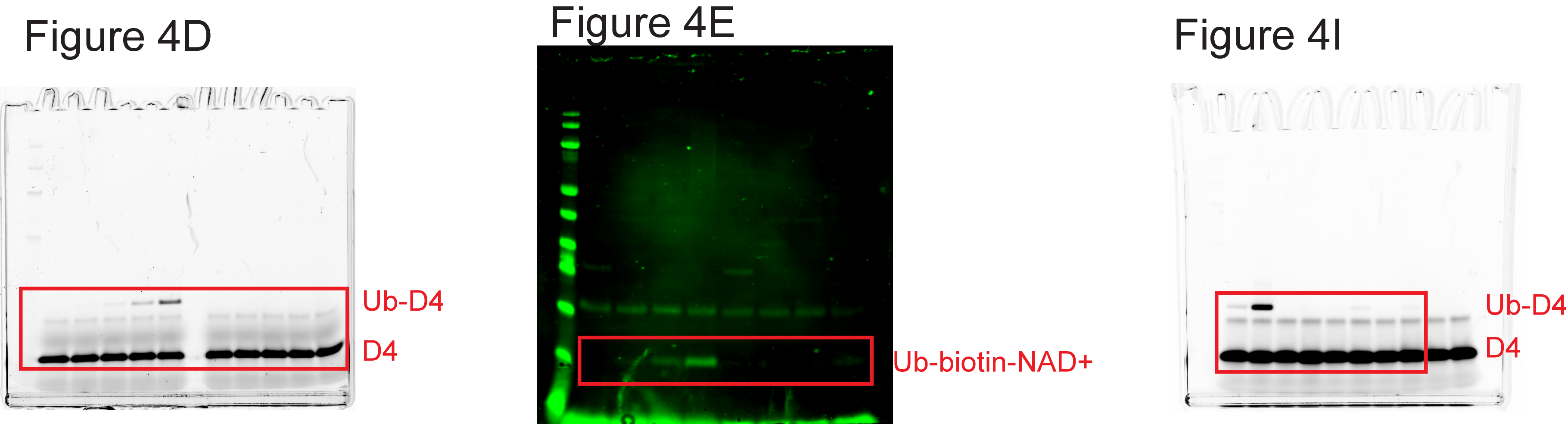

Supplement: Figure 4—source data 2. [file elife-98070-fig4-data2.zip › Figure 4_source data 2/Figure 4_labelled images.tif]

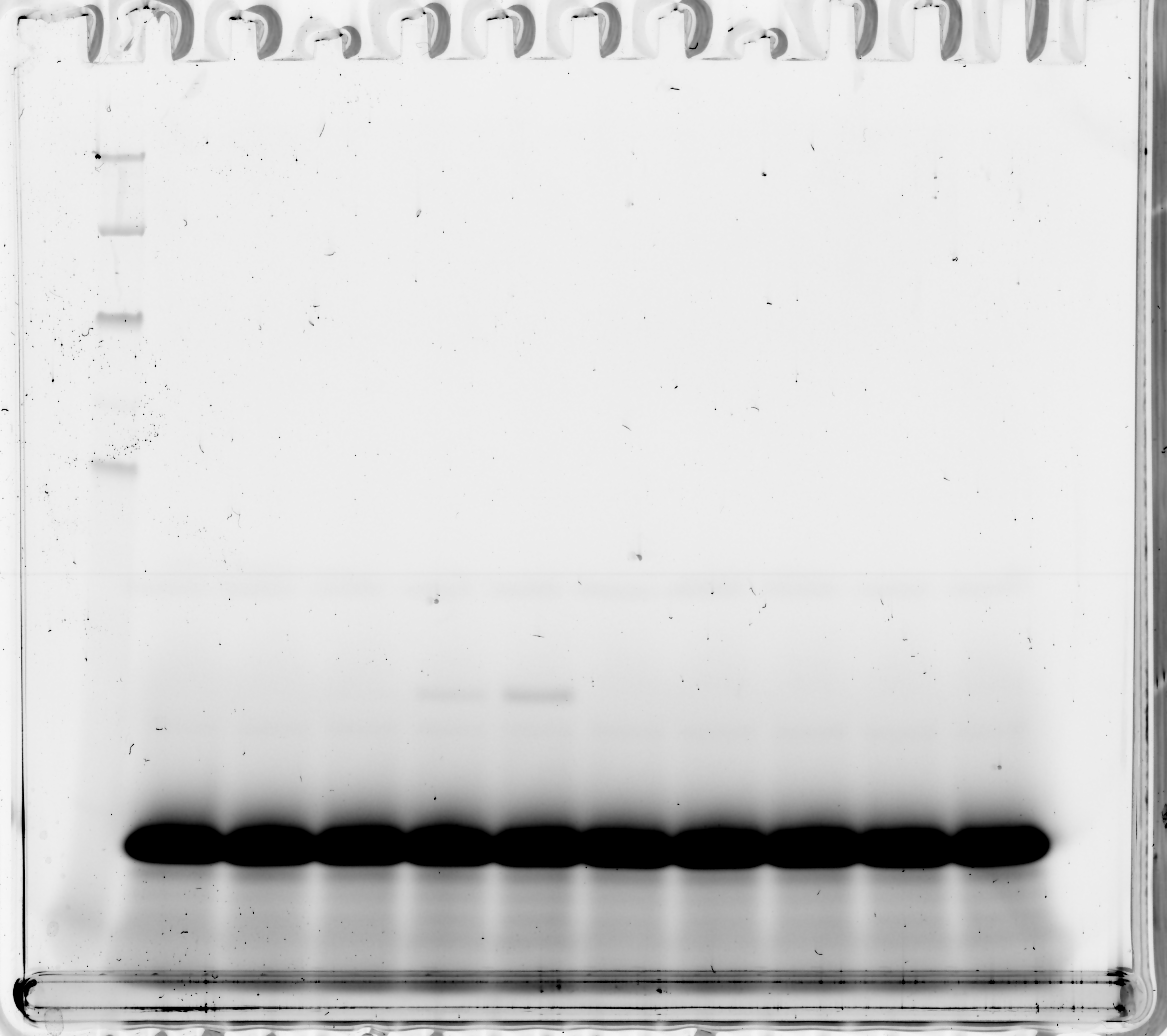

Supplement: Figure 4—figure supplement 1—source data 1. [file elife-98070-fig4-figsupp1-data1.zip › Figure 4_supplementary 1_source data 1/S4C.tif]

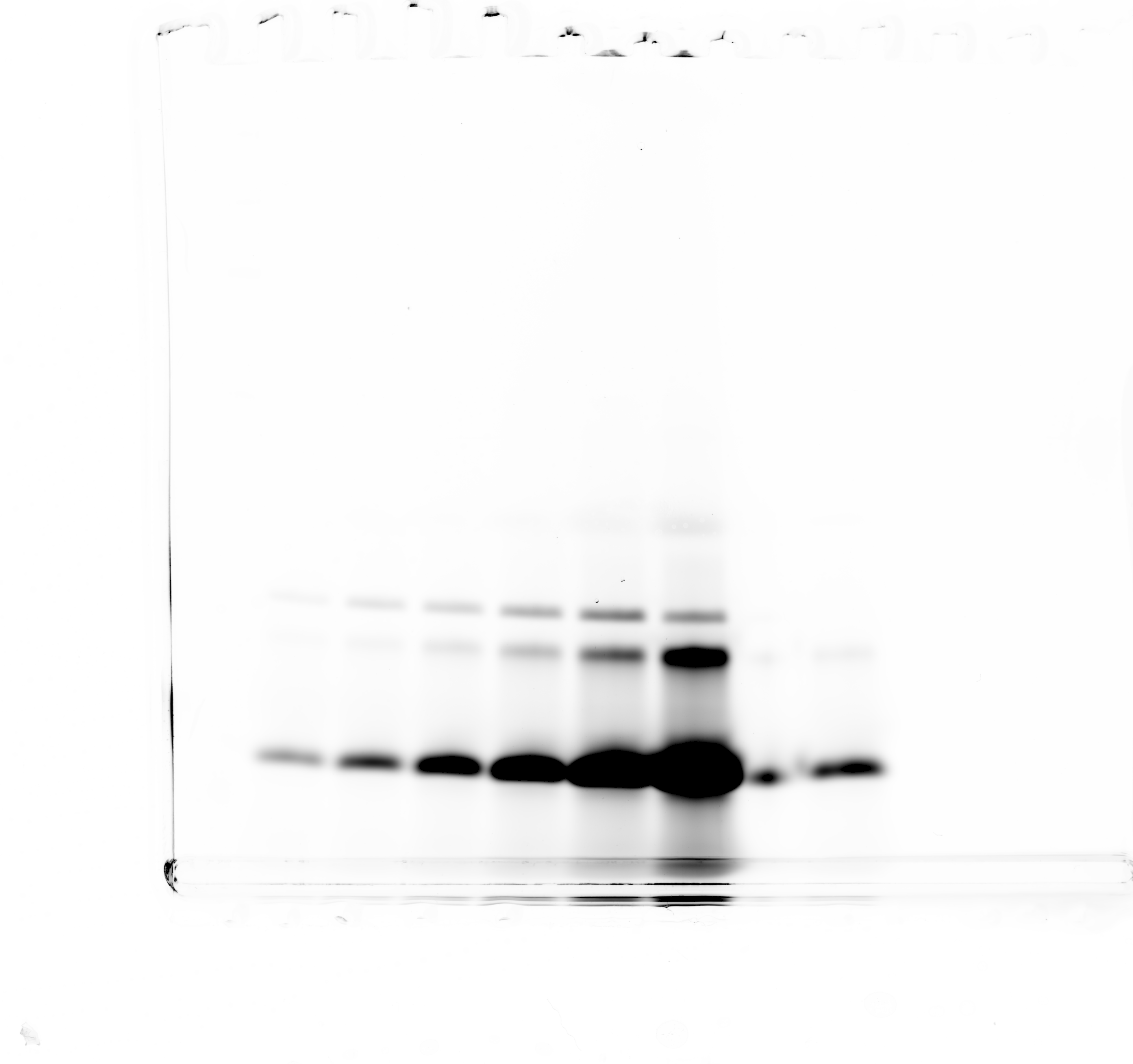

Supplement: Figure 4—figure supplement 1—source data 1. [file elife-98070-fig4-figsupp1-data1.zip › Figure 4_supplementary 1_source data 1/S4D.tif]

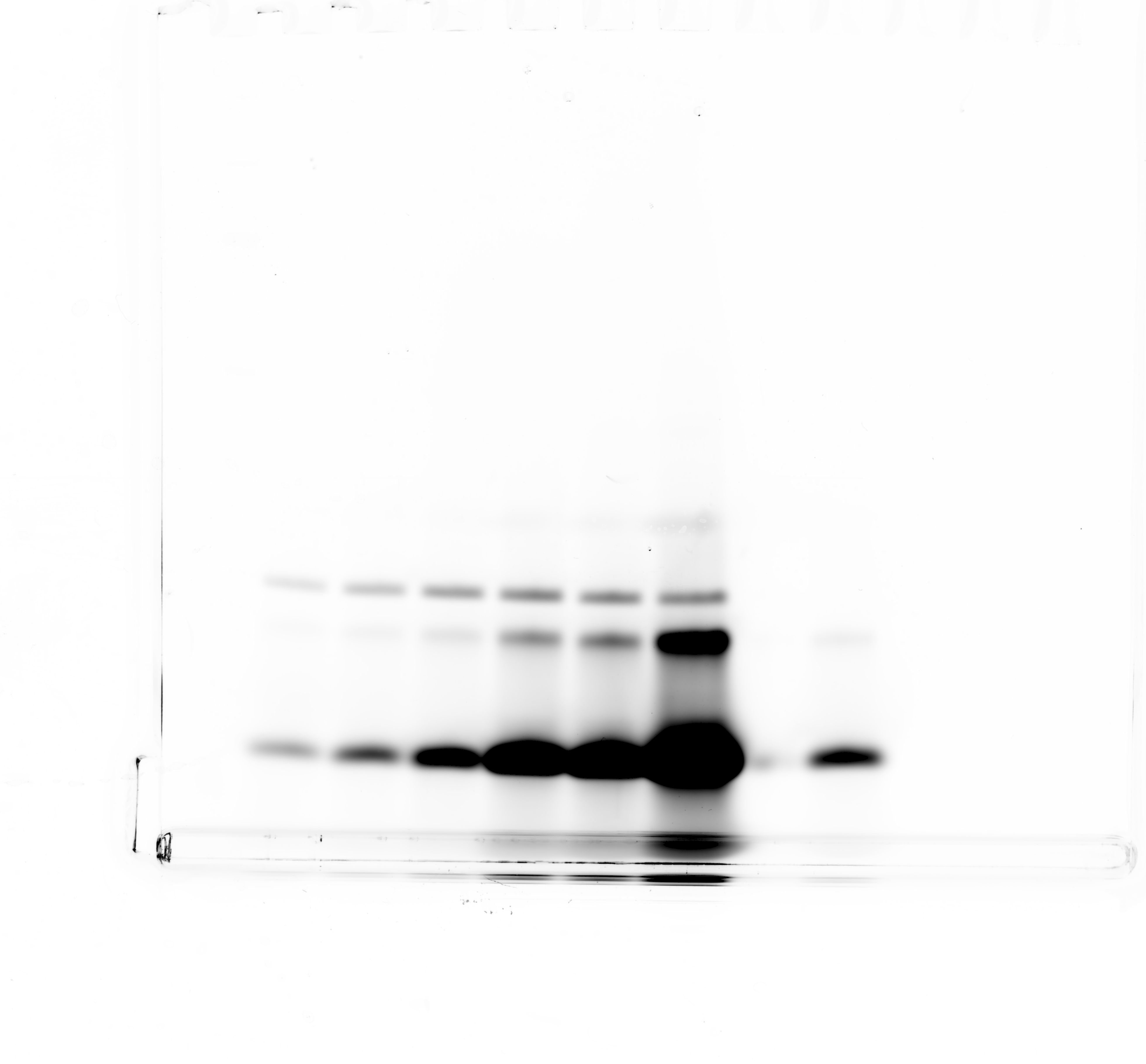

Supplement: Figure 4—figure supplement 1—source data 1. [file elife-98070-fig4-figsupp1-data1.zip › Figure 4_supplementary 1_source data 1/S4E.tif]

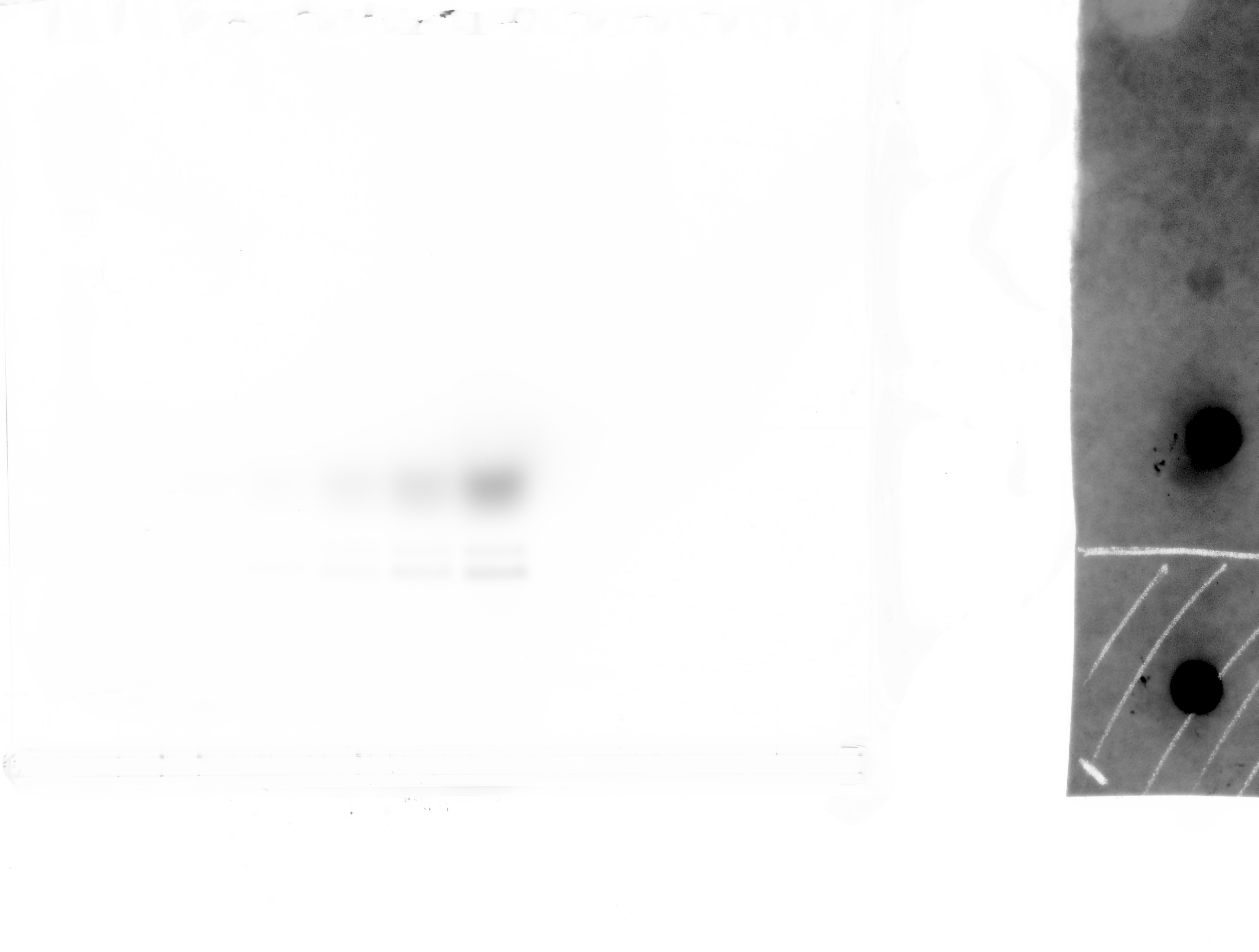

Supplement: Figure 4—figure supplement 1—source data 1. [file elife-98070-fig4-figsupp1-data1.zip › Figure 4_supplementary 1_source data 1/S4F.tif]

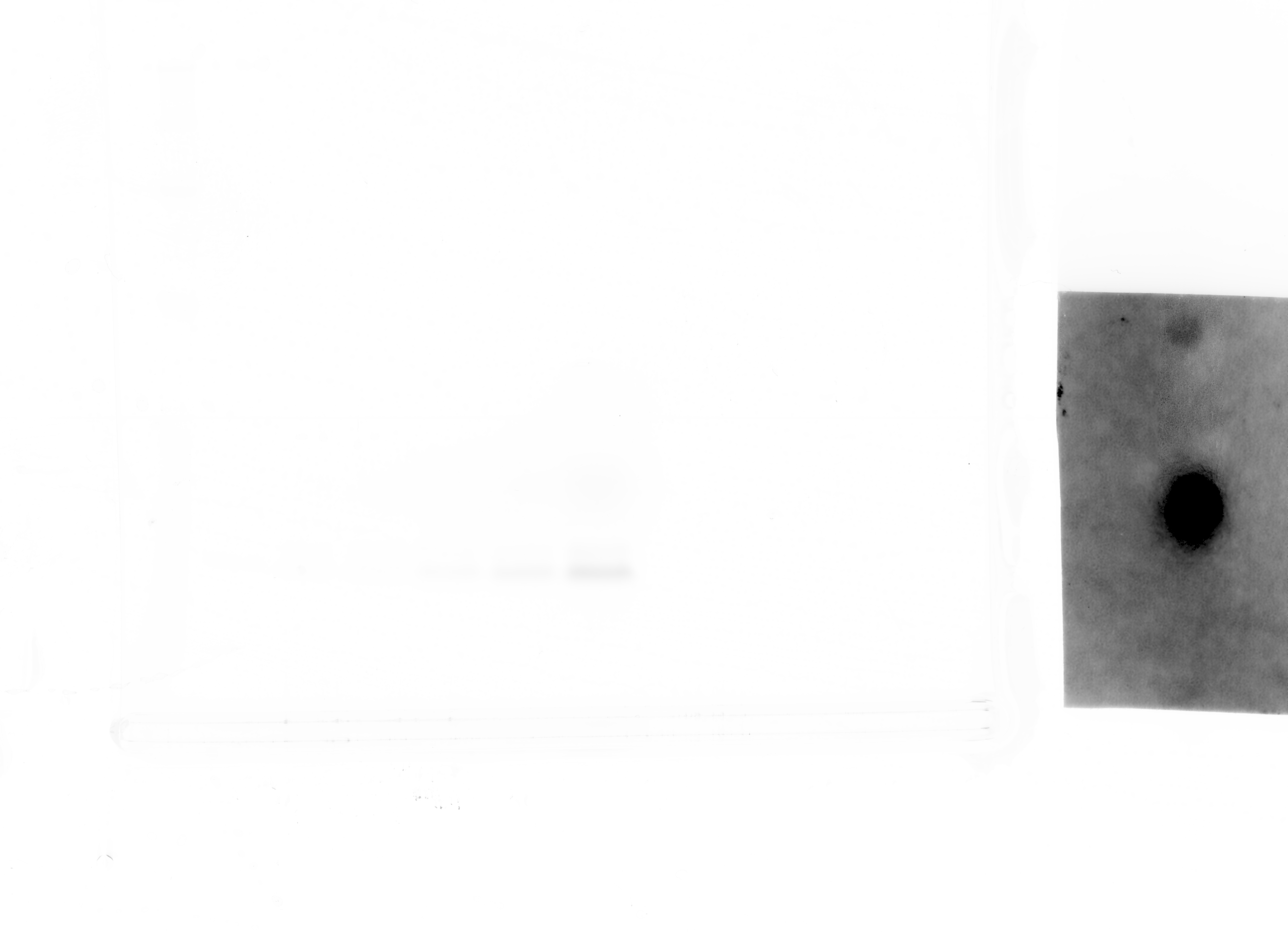

Supplement: Figure 4—figure supplement 1—source data 1. [file elife-98070-fig4-figsupp1-data1.zip › Figure 4_supplementary 1_source data 1/S4G.tif]

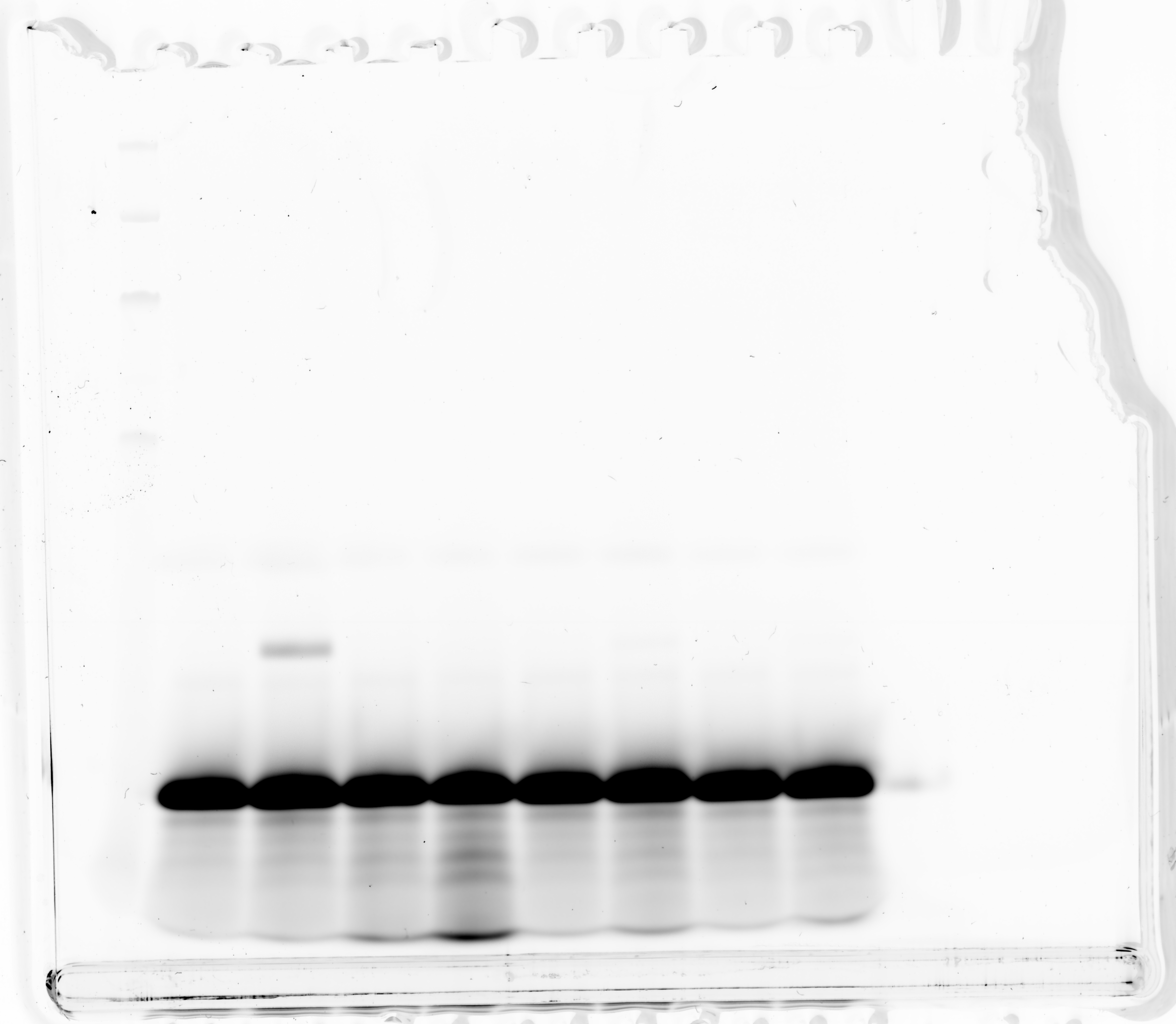

Supplement: Figure 4—figure supplement 1—source data 1. [file elife-98070-fig4-figsupp1-data1.zip › Figure 4_supplementary 1_source data 1/S4H.tif]

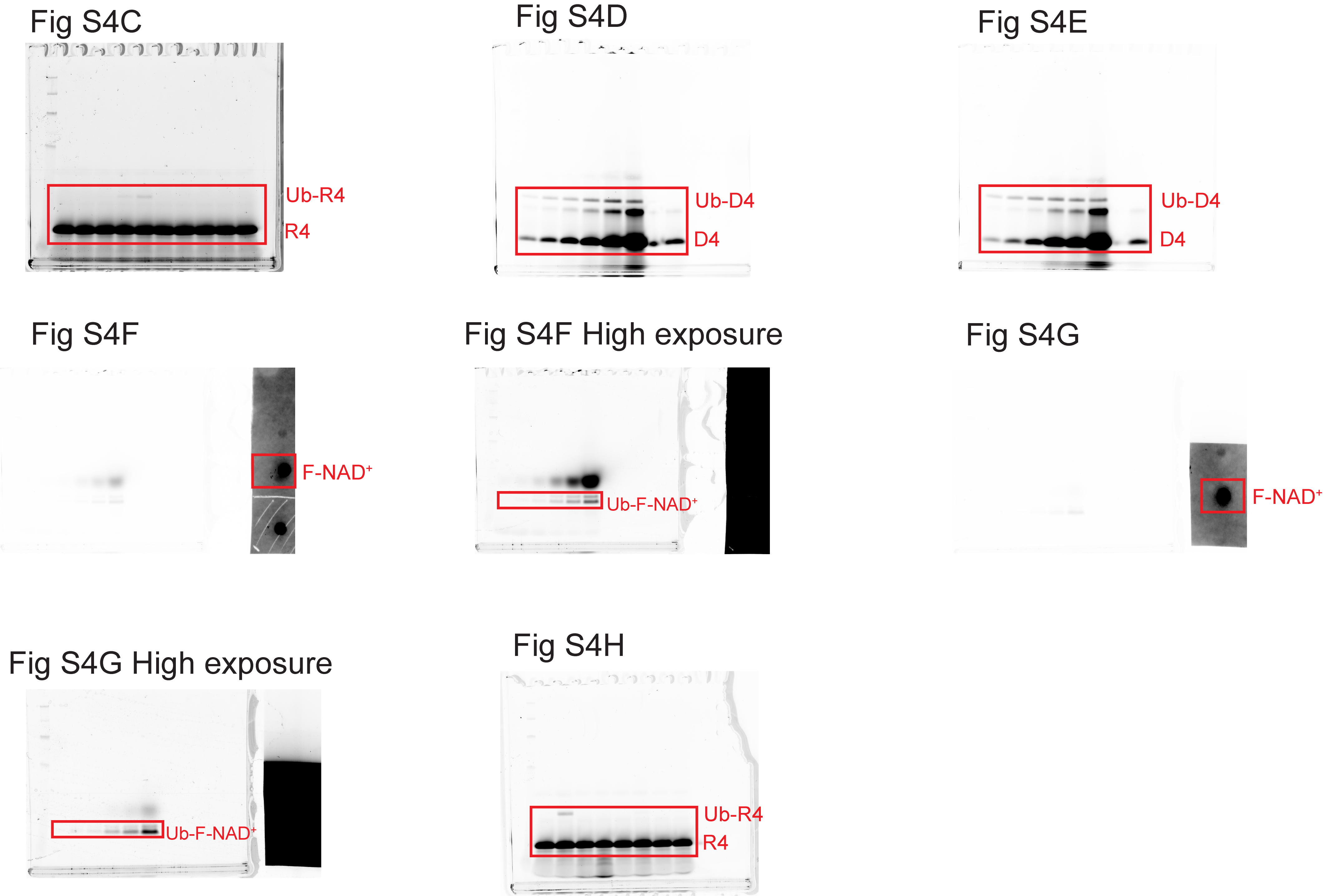

Supplement: Figure 4—figure supplement 1—source data 2. [file elife-98070-fig4-figsupp1-data2.zip › Figure 4_figure supplement 1_source data 2/Figure 4_figure supplement 1_labelled images.tif]

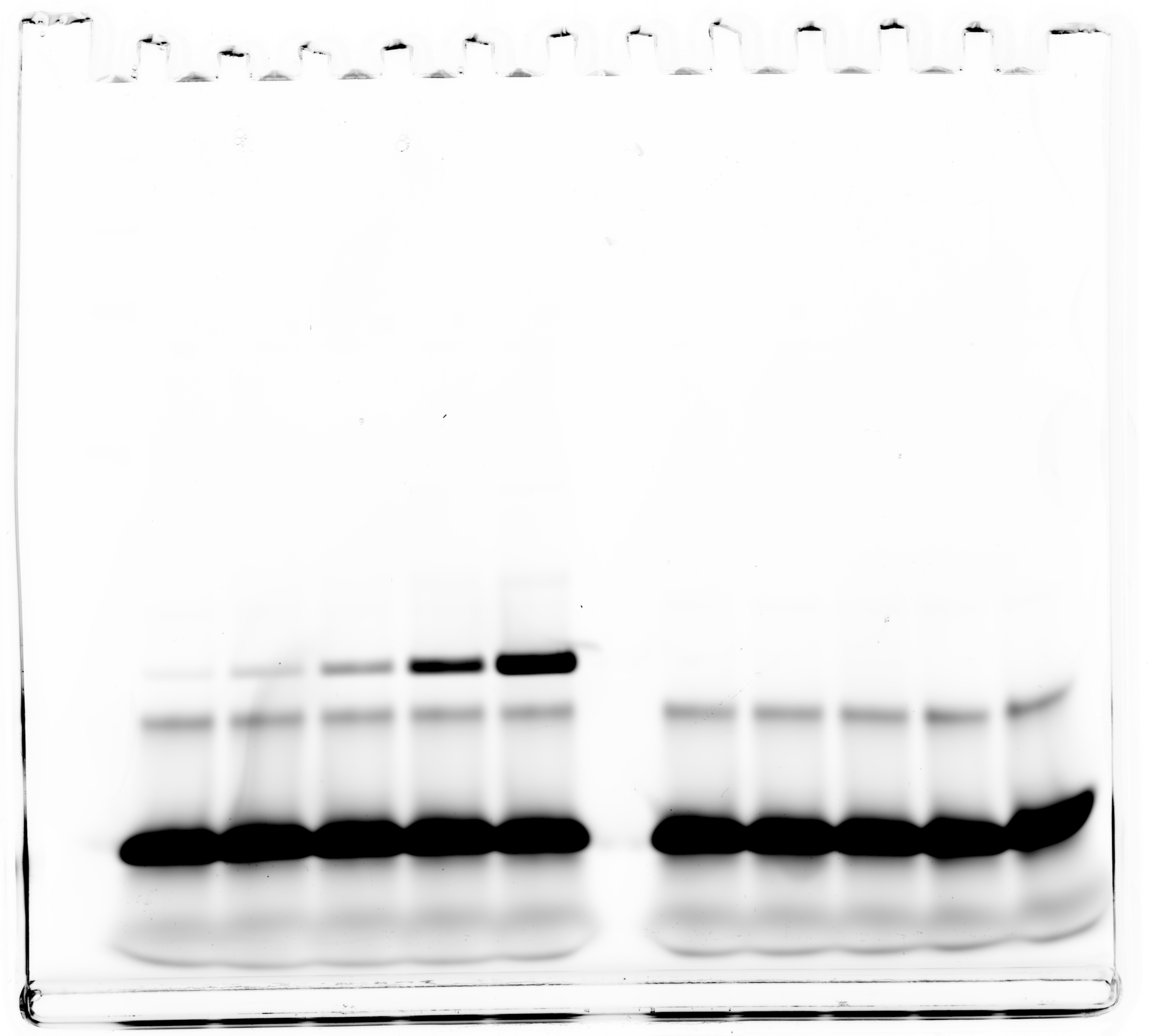

Supplement: Figure 5—source data 1. [file elife-98070-fig5-data1.zip › Figure 5_source data 1/5A.tif]

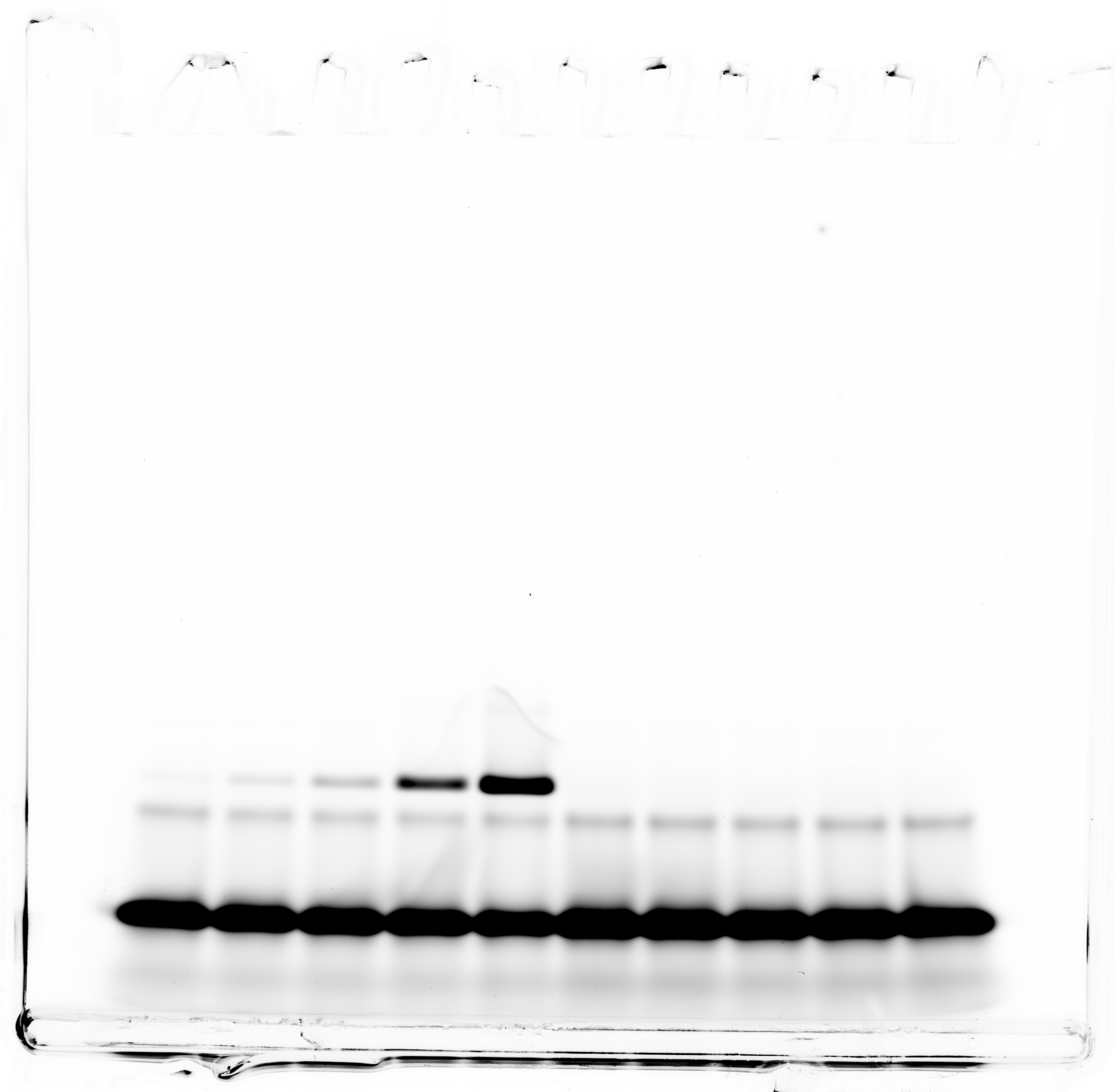

Supplement: Figure 5—source data 1. [file elife-98070-fig5-data1.zip › Figure 5_source data 1/5B.tif]

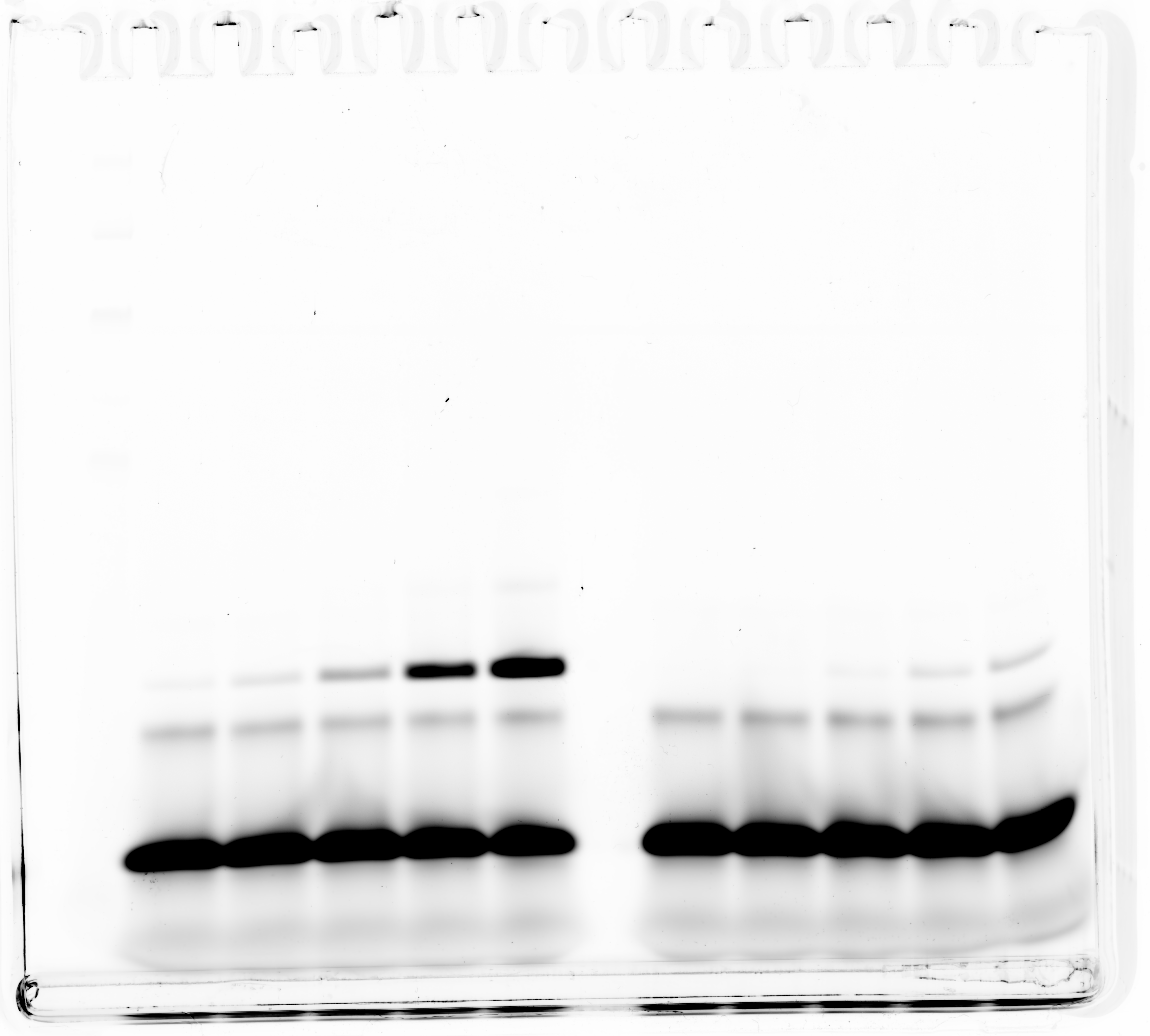

Supplement: Figure 5—source data 1. [file elife-98070-fig5-data1.zip › Figure 5_source data 1/5C.tif]

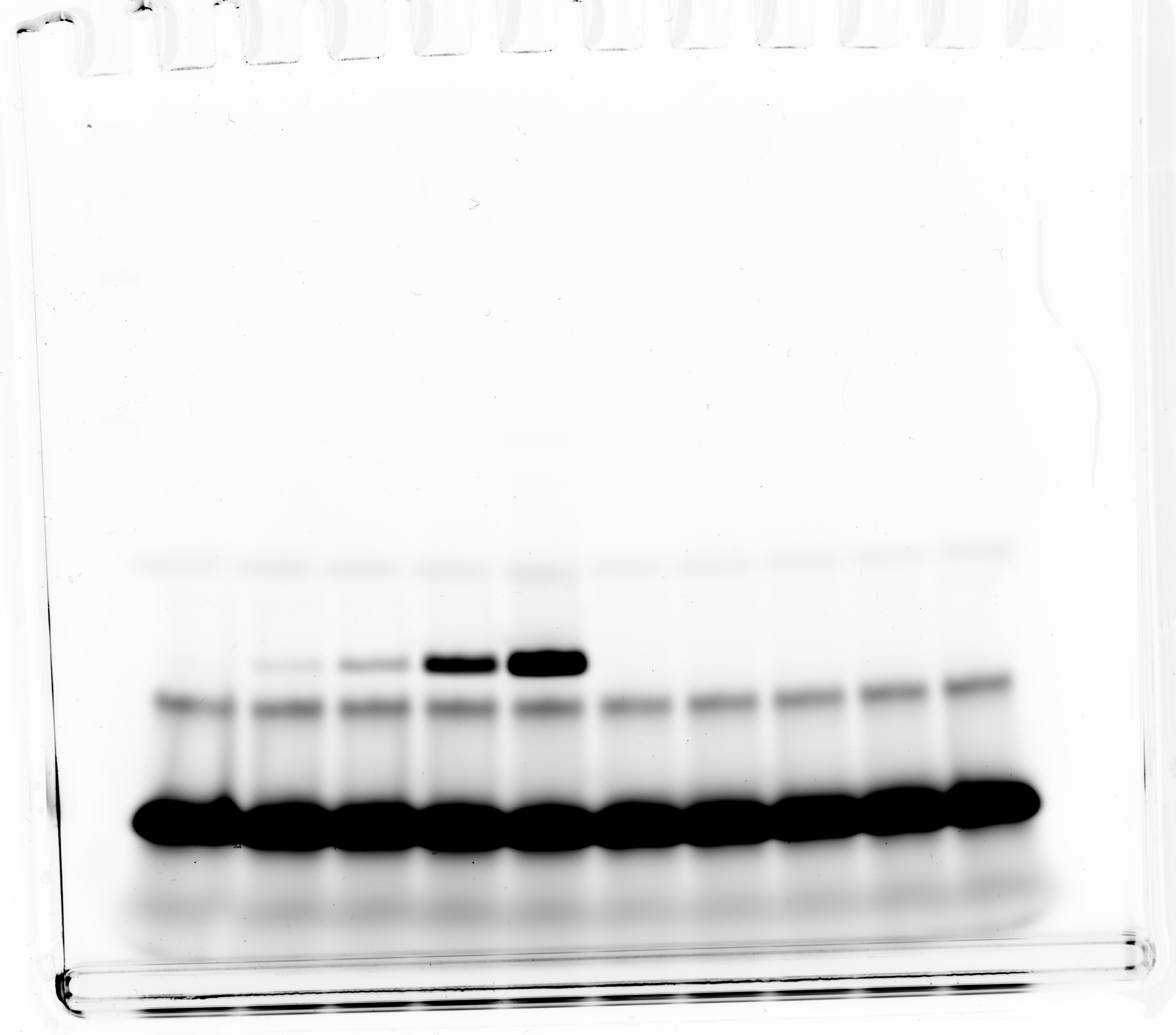

Supplement: Figure 5—source data 1. [file elife-98070-fig5-data1.zip › Figure 5_source data 1/5D.tif]

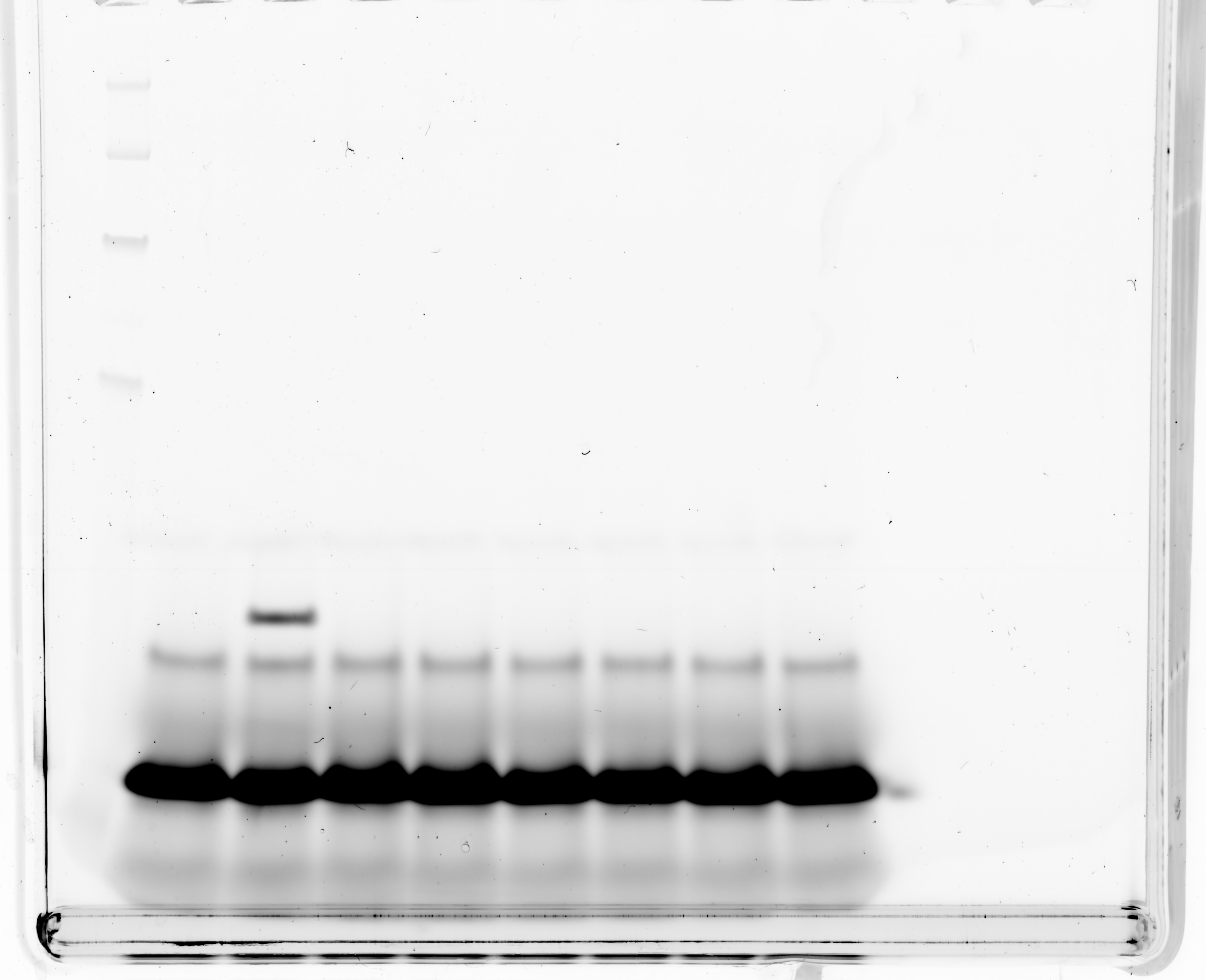

Supplement: Figure 5—source data 1. [file elife-98070-fig5-data1.zip › Figure 5_source data 1/5E.tif]

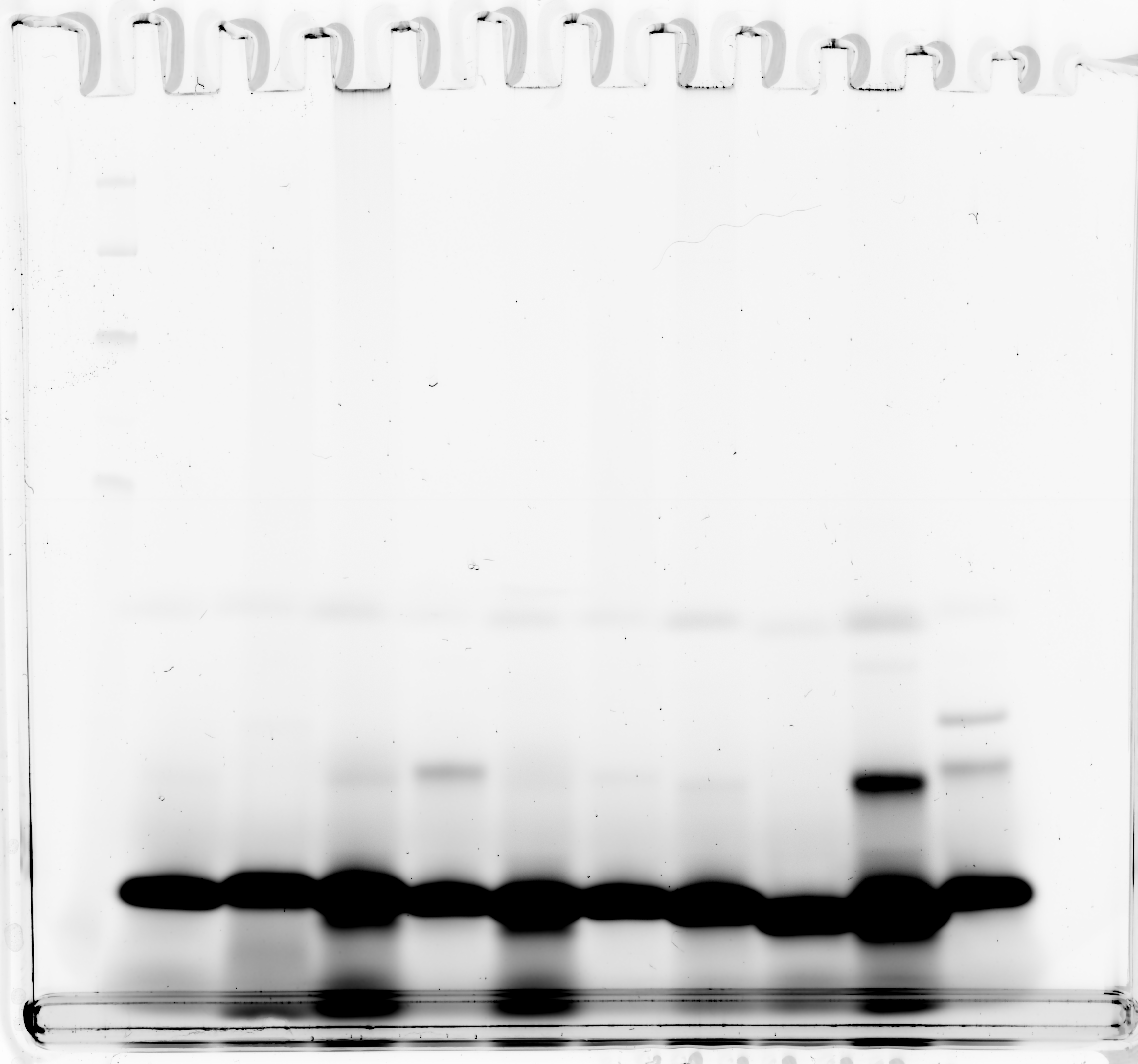

Supplement: Figure 5—source data 1. [file elife-98070-fig5-data1.zip › Figure 5_source data 1/5F.tif]

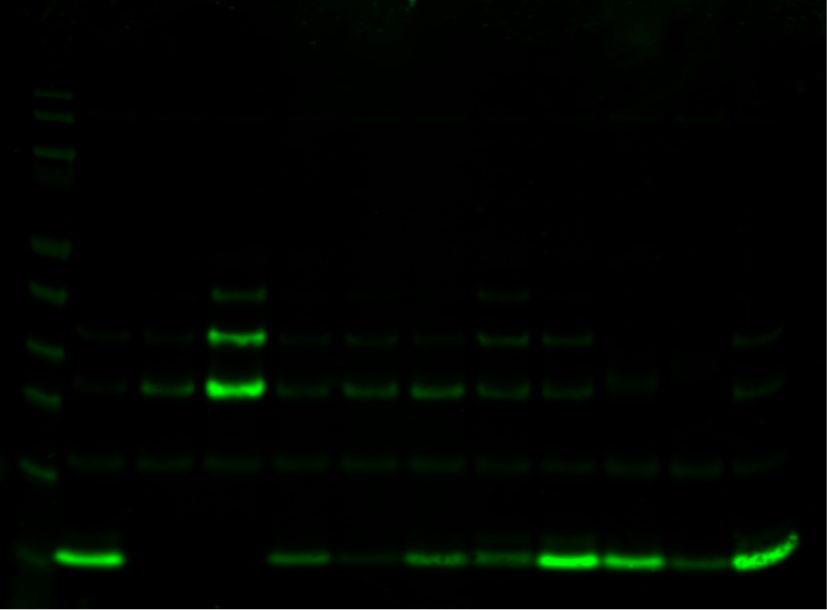

Supplement: Figure 5—source data 1. [file elife-98070-fig5-data1.zip › Figure 5_source data 1/5G.tif]

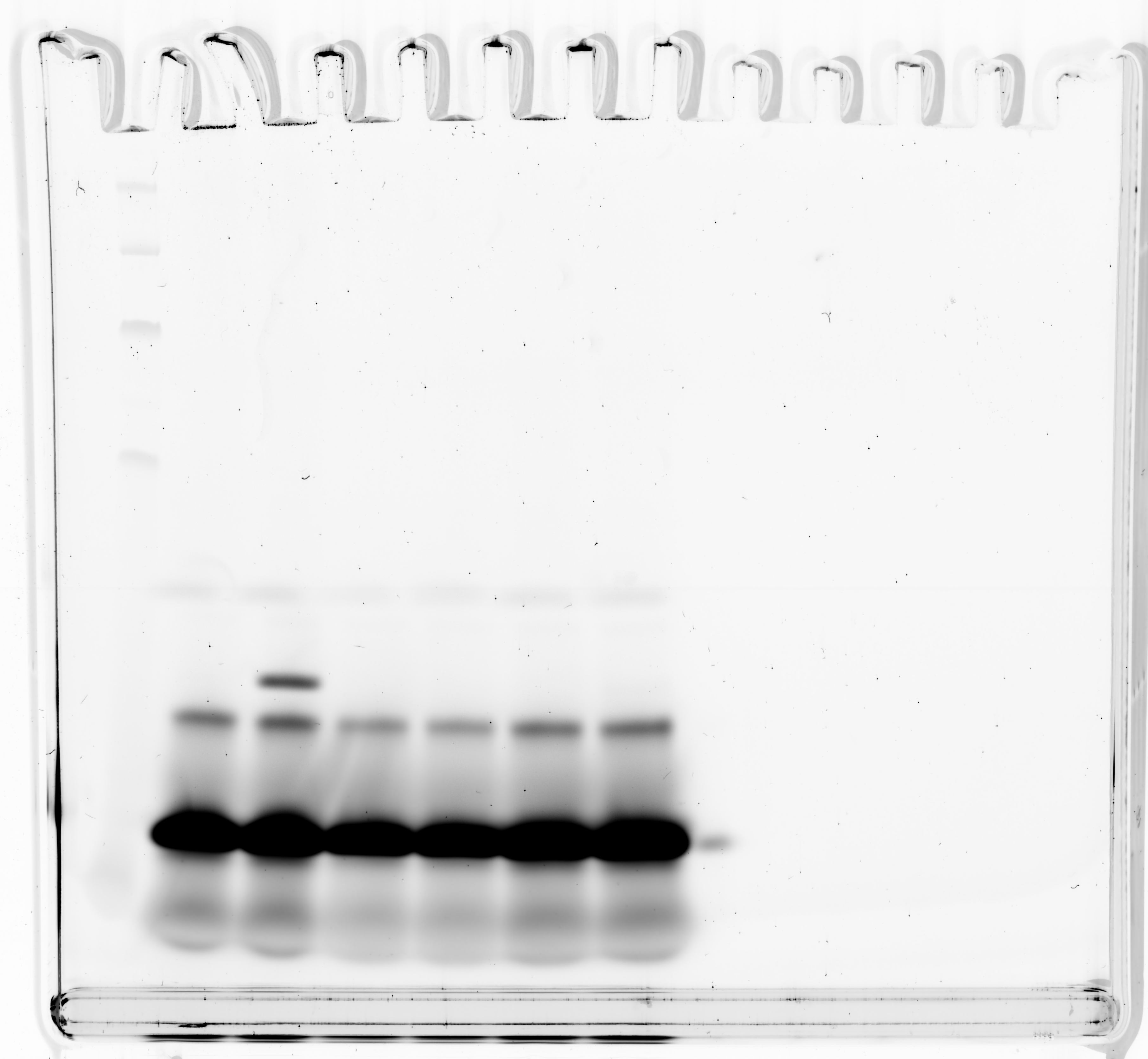

Supplement: Figure 5—source data 1. [file elife-98070-fig5-data1.zip › Figure 5_source data 1/5H.tif]

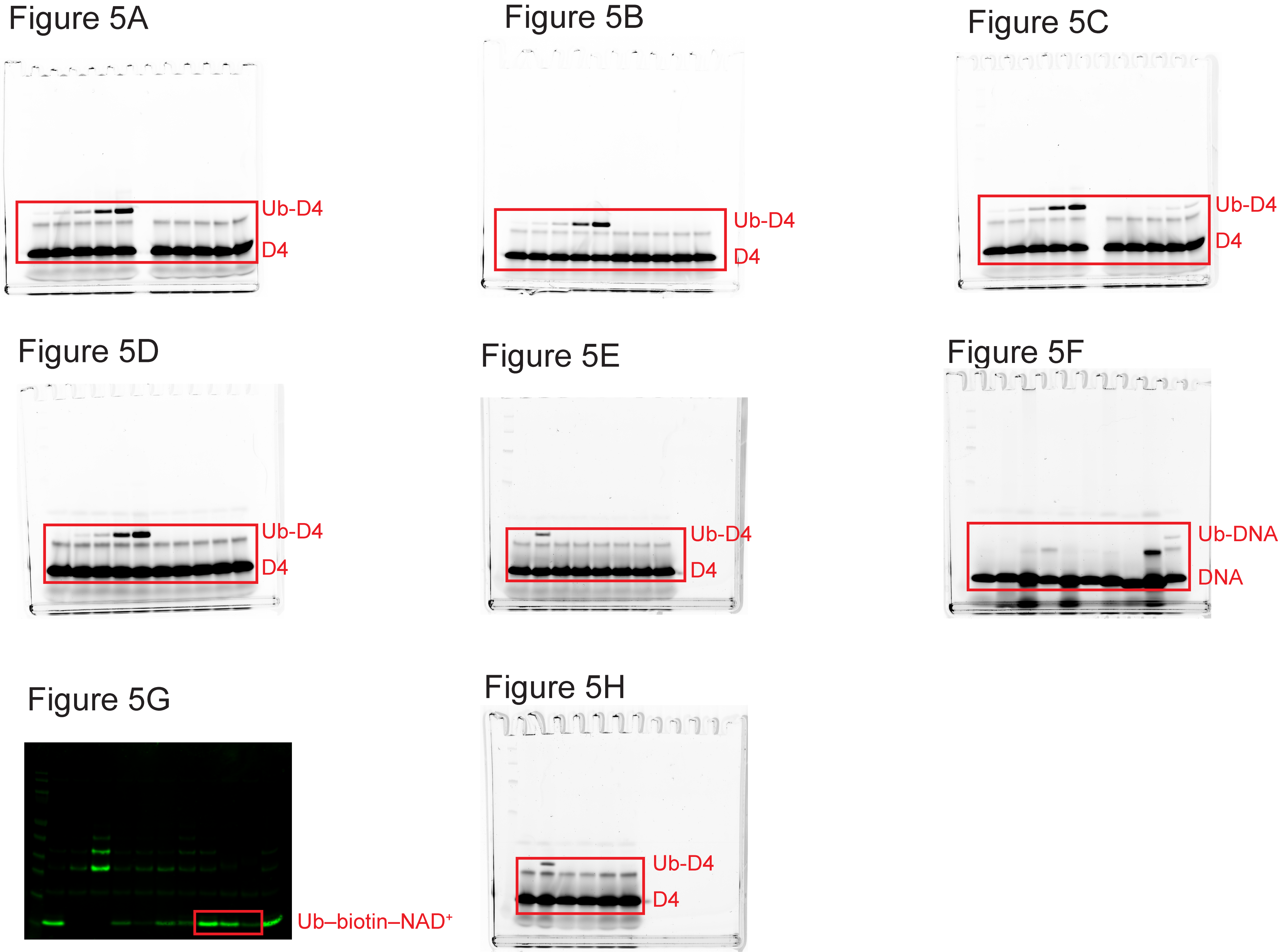

Supplement: Figure 5—source data 2. [file elife-98070-fig5-data2.zip › Figure 5_source data 2/figure 5_labelled images.tif]
